# Inhibition of the CEBPβ-NFκB interaction by nanocarrier-packaged Carnosic acid ameliorates glia-mediated neuroinflammation and improves cognitive function in an Alzheimer’s disease model

**DOI:** 10.1038/s41419-022-04765-1

**Published:** 2022-04-07

**Authors:** Wang Yi-Bin, Li Xiang, Yang Bing, Zhang Qi, Jiao Fei-Tong, Wang Minghong, Zhang Xiangxiang, Kang Le, Li Yan, Sui Ping, Gao Yufei, Xu Ye, Wang Chun-Yan

**Affiliations:** 1 Institute of Health Sciences, Key Laboratory of Medical Cell Biology of Ministry of Education, China Medical University, Shenyang, 110122 China; 2 Translational Medicine Laboratory, School of Basic Medical Sciences, Jilin Medical University, Jilin, 132013 China

**Keywords:** Cognitive ageing, Diseases of the nervous system

## Abstract

Neuroinflammation occurs early in Alzheimer’s disease (AD). The initial stage of AD is related to glial dysfunction, which contributes to impairment of Aβ clearance and disruption of synaptic connection. CEBPβ, a member of the CCAAT-enhancer-binding protein (CEBP) family, modulates the expression of inflammation-associated genes, and its expression is elevated in brains undergoing degeneration and injured brains. However, the mechanism underlying CEBPβ-mediated chronic inflammation in AD is unclear. In this study, we observed that increases in the levels of nuclear CEBPβ facilitated the interaction of CEBPβ with the NFκB p65 subunit, increasing the transcription of proinflammatory cytokines in the APP/PS1 mouse brain. Oral administration of nanocarrier-packaged carnosic acid (CA) reduced the aberrant activation of microglia and astrocytes and diminished mature IL-1β, TNFα and IL-6 production in the APP/PS1 mouse brain. CA administration reduced β-amyloid (Aβ) deposition and ameliorated cognitive impairment in APP/PS1 mice. We observed that CA blocked the interaction of CEBPβ with NFκB p65, and chromatin immunoprecipitation revealed that CA reduced the transcription of the NFκB target genes TNFα and IL-6. We confirmed that CA alleviated inflammatory mediator-induced neuronal degeneration and reduced Aβ secretion by inhibiting the CEBPβ-NFκB signalling pathway in vitro. Sulfobutyl ether-beta-cyclodextrin (SBEβCD) was used as the encapsulation agent for the CA-loaded nanocarrier to overcome the poor water solubility and enhance the brain bioavailability of CA. The CA nanoparticles (NPs) had no obvious toxicity. We demonstrated a feasible SBEβCD-based nanodelivery system targeting the brain. Our data provide experimental evidence that CA-loaded NPs are potential therapeutic agents for AD treatment.

## Introduction

Alzheimer’s disease (AD), the most common cause of dementia, has become one of the most critical global public health issues with the ageing of the population; however, effective interventions are still lacking due to the complexity of the mechanisms underlying AD, including central and peripheral dysfunction. Cytokine dysregulation is considered to play key roles in AD [1, 2]. It has been found that interleukin (IL)-1β [3], IL-6 [4] and transforming growth factor (TGF)-β [5] accumulate around β-amyloid (Aβ) plaques in the AD brain postmortem. A series of clinical studies have indicated that neuroinflammation occurs early in AD [6, 7]. Glial dysfunction occurs in the initial stage of AD [8]. Senescent glia produces more inflammatory cytokines [9]. An imbalance between pro- and anti-inflammatory cytokines is present in the CSF and serum of mild cognitive impairment (MCI) or AD patients. There are significant increases in the levels of proinflammatory cytokines, such as IL-1β, IL-6, and tumour necrosis factor (TNF)-α, and decreases in the levels of anti-inflammatory cytokines, such as the IL-1 receptor antagonist IL-10 [7]. IL-1β elevation can further facilitate the production of IL-6, which may result in an increase in the level of hyperphosphorylated tau, the major component of intracellular neurofibrillary tangles (NFTs) [10]. Moreover, systemic immune challenges triggered by inflammatory inducers can lead to Aβ accumulation and gliosis [11]. In addition, phosphorylated tau can directly bind to the cytokine CX3CL1 receptor, which may provoke microglia, leading to tau internalisation by microglia and the spreading of tau hyperphosphorylation [12].

The incidence of AD is notably decreased among patients with rheumatoid arthritis treated with long-term nonsteroidal anti-inflammatory drug (NSAID) therapy [13, 14], although clinical trials of NSAIDs for AD treatment with small sample sizes failed to provide the expected outcomes [15, 16]. The inconsistency of these findings is probably due to the fact that these anti-inflammatory agents target generic components rather than particular inflammatory molecules associated with AD [17]. Butchart and colleagues obtained positive results when using TNFα inhibitors to treat AD [18]. Targeting the imbalance between pro- and anti-inflammatory cytokines in AD by regulating the homoeostasis of cytokines and proinflammatory genes is a potential therapeutic strategy for AD, and targeting the upstream modulators of cytokines is an attractive approach [18].

The roles of the transcription factor CEBPβ, a member of the CCAAT-enhancer-binding protein (CEBP) family, have attracted increasing attention in regulating cytokine-mediated inflammatory responses. The binding sites in the regions of the CEBPβ promoter and enhancer endow the transcription factor with the ability to regulate a number of cytokines and proinflammatory genes [19]. CEBPβ modulates IL-1β production in macrophages in vitro [20]. Papin and colleagues observed that CEBPβ is an essential regulator that confers cell responsivity to TNFα [21]. CEBPβ knockdown alleviates the expression of proinflammatory genes and reduces the neurotoxic effects of activated microglia [22]. Inhibition of CEBPβ ameliorates glial activation and the neuroinflammatory response in a rat model of Parkinson’s disease [23]. Interestingly, CEBP interacts with NFκB. The C-terminus of the *Rel* homology domain of NFκB can bind with the bZIP region of CEBP, enhancing the transcription of inflammatory genes by NFκB [24]. Importantly, CEBPβ expression in the cortex is markedly increased in specimens from AD patients compared with those from nondemented elderly individuals at autopsy [25]. The transcript levels of *Cebpb* are higher in the brains of aged AD transgenic mice than in the brains of age-matched controls [26]. Downregulating *Cebpb* gene expression ameliorates Tau pathology and cognitive dysfunction in an AD mouse model [27]. Targeting CEBPβ intervention might be a potential therapeutic strategy for mitigating the neuronal damage triggered by inflammation in neurodegenerative diseases.

Carnosic acid (CA), also named (4a*R*,10aS)-5,6-dihydroxy-7-isopropyl-1,1-dimethyl-1,3,4,9,10,10a-hexahydro-*2H*-phenanthrene-4a-carboxylic acid, a phenolic diterpene isolated from rosemary, has attracted increasing attention because of its pharmacologic properties. Through its antiadipogenic activity, CA plays role in inhibiting colon tumour formation [28]. CA can inhibit inflammation and joint destruction in an animal model of arthritis by reducing the levels of TNFα, IL-1β and IL-6 [29]. Interestingly, CA-mediated decreases in glucose levels and diabetic nephropathy remission are involved in inhibition of NFκB [30]. An analogue of CA, Carnosol, could mitigate ROS-mediated bone loss, which was involved in the regulating on NFκB [31]. Oxidative stress and inflammation are linked. Inflammation could interact with oxidising agents, triggering oxidative stress. Accordingly, oxidative stress facilitates the release of inflammatory cytokines [32]. It is reasonable to speculate that CA may play a neuroprotective role in modulating the CEBPβ-NFκB-cytokine network in AD.

In this study, we investigated the effects of CA treatment on pro- and anti-inflammatory cytokine homoeostasis and the potential effects of CA on CEBPβ target genes and molecular pathways in the treatment of AD. The lipophilicity and poor water solubility of CA and the feasibility of delivering CA to the brain should be considered. Furthermore, the acceptance of oral noninvasive therapy is high, but the first-pass effect of the liver and the influence of the gastrointestinal environment on drugs should also be considered. Here, we developed a sulfobutyl ether-beta-cyclodextrin (SBEβCD)-based nanoparticles (NPs) drug carrier that can deliver CA to the brain. We observed that CA suppressed CEBPβ transcriptional activity and reduced the interaction between CEBPβ and NFκB in the brains of APP/PS1 Tg mice. CA-mediated decreases in CEBPβ expression inhibited amyloidogenesis. Our results reveal the therapeutic effects of CA on the CEBPβ network and show that CA might be a promising therapeutic agent for the treatment of AD.

## Materials and methods

### Preparation of SBEβCD and CA-*SBEβCD NPs*

SBEβCD (1.05 g) was dissolved in 5 mL of double-distilled water. The solution was then added to 1.0 mL of Tween 80, 10.0 mL of 10% poloxamer 407 and 2.0 mL of polyethylene glycol 400. After vortexing, the emulsion was used as the solvent for SBEβCD nanoparticles (*SBEβCD NPs*). The pH was adjusted to 7.2 using trihydroxymethyl aminomethane. *SBEβCD NPs* containing CA (purity, 99.034%; Shanghai Winherb Medical Science Co., Ltd., Shanghai, China), namely, *CA-SBEβCD NPs*, were prepared as follows: CA (0.24 g) was dissolved in 0.8 mL of 100% ethanol. Under continuous magnetic stirring, CA-ethanol solution was gradually added to the SBEβCD inclusion complex (IC) (drug-SBEβCD molar ratio, 1:1) [33] at room temperature, and the total volume was adjusted to 40.0 mL with deionized water. After ultrasonication (500 W, 3 s interval, 15 min), the formed NPs were filtered through a 0.22-μm microporous membrane.

### Characterisation of the NP system

#### Size distribution, surface charge and morphology of the NPs

The particle size and zeta potentials were measured by laser diffraction or Doppler velocimetry at 25 °C with a Zetasizer Nano ZS90 instrument (Malvern Instruments Ltd., Malvern, Worcestershire, UK). All measurements were conducted at least three times.

After negative staining using 1% phosphotungstic acid, the morphology of the NPs was examined with a transmission electron microscope (TEM) (JEM-2100, Nikon, Japan) at an accelerating voltage of 200 kV.

#### Encapsulation and loading efficiency assays

*CA-SBEβCD NPs* were assessed by high-performance liquid chromatography (HPLC). Briefly, to separate the free CA from the *CA-SBEβCD NPs*, 5 mg of lyophilised NPs containing CA were dissolved in 1 mL of dichloromethane. The organic phase was then collected and extracted using 5 mL of ethanol. After centrifugation, the supernatants were filtered with a microporous membrane for HPLC. The chromatographic conditions were as follows: Phenomenex C18 analytical column (150 mm × 4.6 mm, 5 micron particle size); mobile phase, acetonitrile-0.1% phosphoric acid (55:45, v/v); column temperature, 25 °C; flow rate, 1.0 mL/min; and injection volume, 20 μL. The encapsulation efficiency (EE) and loading efficiency (LE) were calculated as follows:

$${{{\mathrm{Encapsulation}}}}\;{{{\mathrm{efficiency}}}}\,\left( {{{\mathrm{\% }}}} \right) = \frac{{{\mathrm{mass}}\;{\mathrm{of}}\;{\mathrm{the}}\;{\mathrm{CA}} - {\mathrm{unbound}}\;{\mathrm{CA}}}}{{{\mathrm{mass}}\;{\mathrm{of}}\;{\mathrm{the}}\;{\mathrm{CA}}}} \times 100$$

$${{{\mathrm{Loading}}}}\;{{{\mathrm{efficiency}}}}\,\left( {{{\mathrm{\% }}}} \right) = \frac{{{\mathrm{mass}}\;{\mathrm{of}}\;{\mathrm{the}}\;{\mathrm{CA}} - {\mathrm{unbound}}\;{\mathrm{CA}}}}{{{\mathrm{mass}}\;{\mathrm{of}}\;{\mathrm{the}}\;{\mathrm{nanoparticles}}}} \times 100$$

#### Hydrogen nuclear magnetic resonance (^1^H-NMR) spectroscopy

The chemistry of the NP system was evaluated with a ^1^H-NMR spectrometer (Bruker AVANCE III HD 500 MHz, Switzerland). Chloroform-d (deuterochloroform, CDCL 3) was used as the solvent. Hydrogen spectrum data were analysed and plotted with MestReNova combined with Origin software.

#### In vitro drug release analysis

Dynamic dialysis was performed to assess CA release from the *CA-SBEβCD NP*s. Briefly, 2 mL of *CA-SBEβCD NPs* was transferred into a cellulose-based dialysis tube (molecular weight cut-off: 14,000 Da) that had been pre-soaked in distilled water. The tube was closed tightly, placed into an oscillator containing 50 mL of 0.01 M phosphate-buffered saline (PBS, pH 7.4 and pH 6.0) and gently shaken at 100 rpm (37 °C). Five millilitres of the medium was assessed at the indicated times (0.25, 0.5, 1, 2, 4, 6, 8, 10, 24, 48 and 72 h), and an equal volume of PBS at the same temperature was added at each sampling time. The amount of CA in the sample at each time point was determined by HPLC according to the chromatographic conditions above, and the cumulative release percentage was calculated.

#### Safety evaluation of the NPs

To assess the cytotoxicity of the nanosystem in vitro, the NP system was administered to human neuroblastoma SH-SY5Y cells. SH-SY5Y cells were routinely plated in 96-well plates in Dulbecco’s modified Eagle’s medium (DMEM) containing 10% heat-inactivated foetal bovine serum (FBS), 100 U/mL penicillin and 100 μg/mL streptomycin in a fully humidified incubator with 5% CO_2_ at 37 °C. The cells were maintained in serum-free DMEM until they reached 80% confluence. Two hours later, the cells were exposed to *SBEβCD NPs*, *CA-SBEβCD NPs* and CA as indicated. Untreated cells were used as matched controls. Cell viability was determined by the cell counting kit-8 (CCK-8) assay. The absorbance was measured at 450 nm using a 96-well plate reader.

To assess acute toxicity in vivo, two-month-old male C57BL/6 mice (body weight: 20–22 g), were randomly divided into three groups: orally gavaged with saline, *SBEβCD NPs* or *CA-SBEβCD NPs* (*n* = 8 in each group) every day for 7 days. Animal usage was in accordance with the guidelines established by the Ministry of Health, Peoples Republic of China and the ethical standards for laboratory animals of China Medical University. On the 8th day, the mice were sacrificed. The stomach, jejunum, ileum and colon of each mouse were collected and fixed in 4% paraformaldehyde for 48 h at 4 °C. After dehydrated, 10-µm sections of the tissues were prepared with a freezing microtome. The sections were mounted onto slides and stained with haematoxylin and eosin (H&E).

To evaluate chronic toxicity, male C57BL/6 mice (*n* = 8 in each group) were gavaged with the above-mentioned solutions for 30 days. The body weight of the mice was monitored each day. On day 31, the heart, liver, spleen, lung and kidney were removed. After fixation and dehydration, the samples were sectioned at a thickness of 10 μm and stained with H&E. The operators were blinded to the group allocation during the experiments.

#### Assessment of the BBB permeability of NPs in vitro

Transwell assays were performed to evaluate the ability of the NPs to cross the blood-brain barrier (BBB) and target neuronal cells. Briefly, hCMEC/D3 cells were loaded in the upper chamber of a Transwell system (Corning, NY, USA). The cells were cultured in DMEM containing 10% heat-inactivated FBS, 1% L-glutamic acid and 5 µM 2-mercaptoethanol in a fully humidified incubator with 5% CO_2_ (37 °C). SH-SY5Y cells were seeded in the bottom chamber. The SH-SY5Y cells were cultured in the same manner as hCMEC/D3 cells, but the culture medium was supplemented with 100 U/mL penicillin and 100 μg/mL streptomycin. The medium of both cell types was replenished each day. The transendothelial electrical resistance (TEER) was monitored on consecutive days. After they formed a tight confluent monolayer, the hCMEC/D3 cells in the upper chamber were treated with 6 μM 6-coumarin (Cou6) suspension, nanoemulsion (NE)-dissolved Cou6 or Cou6-tagged *SBEβCD NPs* for 24 h. The SH-SY5Y cells in the bottom chamber were collected and analysed by flow cytometry (FCM).

#### Evaluation of Cou6-loaded NP localisation in vivo

To investigate the retention and localisation of the drug delivery system, C57BL/6 mice were gavaged with Cou6-loaded *SBEβCD NPs* or NE-dissolved Cou6 (60 mg/kg). After 90 min, the mice were anaesthetised with pentobarbital sodium salt (70 mg/kg) by intraperitoneal injection and sacrificed by decapitation. The liver, kidney and brain of each animal were collected and cut into frozen sections (10 μm), and fluorescence was assessed using a confocal laser scanning microscope (Nikon 1, Japan).

#### Mice and treatment

Male APP/PS1 (B6C3-Tg [APPswe, PSEN1dE9] 85Dbo/Mmjax) double Tg mice (RRID: MMRRC_034829-JAX) and age-matched C57BL/6 mice were obtained from Jackson Laboratory. All animal experimental procedures were approved by the Ethics Committee of China Medical University. Four-month-old mice were randomly assigned to three treatment groups: the group treated with vehicle control (*SBEβCD NPs*), the group treated with *CA-SBEβCD NPs* at a dose of 10 mg/kg CA and the group treated with *CA-SBEβCD NPs* at a dose of 30 mg/kg CA (*n* = 8 in each group). The mice were given the indicated treatment by oral gavage once *per* day for 5 months. The body weights of the mice were recorded, and the general behaviours of the mice were monitored daily.

#### Analysis of cognition

To investigate affiliative social behaviour, the nest construction test was conducted. The mice were housed individually in cages with 1-cm-deep corncob bedding one day before the test. Eight pieces of paper (5 cm × 5 cm) were neatly placed on the bedding to introduce nesting conditions. The nesting behaviour of each mouse was observed the following morning. The nests constructed from the pieces of paper were photographed for seven consecutive days. The nesting scores of the mice were determined according to a four-point scoring system: (1) the paper was not obviously bitten or torn and was randomly scattered; (2) the paper was gathered in a corner or on the side of the cage but not obviously bitten or torn; (3) the paper was gathered in a corner or on the side of the cage and showed moderate biting or tearing; (4) the paper was gathered in a corner or on the side of the cage and showed extensive biting or tearing.

To evaluate the recognition memory of the mice, the novel object recognition test (NORT) was performed. Briefly, the mice were allowed to habituate to the arena (50 cm length × 50 cm width × 40 cm height) and the testing room for 10 min twice a day for 2 days. On the next day, two plastic blocks of the same volume were placed in the arena. Each mouse was given 5 min to explore the blocks. Then, the mouse was placed back in its cage for 5 min, after which it was subjected to a 5-min short-term memory (STM) test. During the STM test, the mouse was placed in the arena after one of the objects was replaced with a novel plastic cylinder. For the long-term memory (LTM) assessment, the familiar or novel objects were replaced, and the interval was 24 h. The exploratory behaviour was defined as when a mouse directed its nose toward an object within a range of 2 cm and/or directly touched the object with its nose and/or forepaws. The discrimination index was calculated as the percentage of time spent exploring the novel object compared to the total time spent exploring both objects.

To investigate the spatial learning and memory of the mice, the Morris water maze (MWM) test was conducted. The test involved 8 days of testing. The mice were allowed to swim in a tank of water. First, the mice underwent 2 days of training in which a visible platform was present in the tank. Then, the mice underwent the navigation test, in which they searched for a hidden platform 1 cm below the water surface, for 5 days. The mice were subjected to three tests, each of which lasted a maximum of 60 s, at a 60-s intertrial interval. The escape latency was recorded using an overhead video tracking system. Finally, on the 8th day, the platform was removed from the tank, and a probe trial was carried out to evaluate the search bias of the mice. The times of each mouse crossed the region in which the platform had previously been located and the distances travelled in this region were recorded.

#### Preparation of tissue samples

Twenty-four hours after the behavioural tests, the mice were deeply anaesthetized using sodium pentobarbital (60 mg/kg, intraperitoneally). After transcardial perfusion with precooled 0.9% saline, the animals were sacrificed by decapitation. The mouse brains were sagittally cut in half. The right hemisphere of each mouse was fixed, dehydrated and cut into frozen sections for morphological analyses. The left hemisphere of each mouse was used to isolate cells or snap-frozen in liquid nitrogen and stored at 80 °C for biochemical assays.

#### Cell culture and treatment

Human glioblastoma A172 cells were cultured in DMEM/F12 (Life Technologies) containing 10% FBS and 1% penicillin/streptomycin. Human neuroblastoma SH-SY5Y cells were obtained from ATCC (ATCC^®^ CRL2266™), which were authenticated (underwent STR analysis, sterility test and Human pathogenic virus testing) and tested for mycoplasma contamination. Cells stably transfected with the human β-amyloid precursor protein Swedish mutation (APPswe) or empty vector (*neo*) were grown in DMEM/F12 containing 10% FBS and 1% penicillin/streptomycin and supplemented with 200 µg/mL G418. When the cells reached 80% confluence, they were cultured in DMEM/F12 without serum, penicillin/streptomycin or G418. lipopolysaccharide (LPS), a major outer surface membrane component of all gram-negative bacteria, was administered to A172 cells to trigger proinflammatory responses. The cells were then incubated with CA, or a lentivirus expressing CEBPβ as indicated.

#### FCM

To assess the degree of mitochondrial membrane polarisation, cultured cells were labelled with 2 μM of JC-1 (MitoProbe^TM^ JC-1 Assay Kit) for 20 min at 37 °C and protected from light. The JC-1 Green (FL1) and JC-1 Red (FL2) signals were quantified using detectors. The ratio of JC-1 red fluorescence signal to JC-1 green fluorescence signal was determined.

For apoptosis evaluation, cells isolated from the prefrontal cortex of the brains were harvested by centrifugation at 400 × *g*, trypsinized, and resuspended in PBS. Cells isolated from the tissue specimens or cultured cells were then fixed in precooled 75% ethanol for 1 h. Apoptotic cells were quantified with an Annexin V-fluorescein isothiocyanate (FITC)/propidium iodide (PI) apoptosis detection kit according to the manufacturer’s instructions. Briefly, the cells were treated with binding buffer (5 µL Annexin V-FITC and 5 µL PI) for 15 min at room temperature in the dark. The samples were analysed by FCM. Apoptosis was quantified using FITC (FL1) and PI (FL2) fluorescence detectors. At least three independent experiments were performed.

#### Immunostaining

Frozen mouse brains were cut coronally into ten-micrometre-thick sections. The sections were rehydrated in 0.01 M PBS and treated with 3% hydrogen peroxide to eliminate endogenous peroxidase activity. The samples were rinsed and then blocked with 5% normal donkey serum for 30 min at room temperature. The sections were incubated with a primary antibody for immunohistochemistry (IHC) or a mixture of primary antibodies for double immunofluorescence (IF) for 16 to 18 h in a humidified chamber at 4 °C. After being thoroughly rinsed, the sections for IHC were incubated with an appropriate biotinylated secondary antibody at room temperature for 2 h and then treated with streptavidin peroxidase for 30 min. After being rinsed, the samples were stained with 0.025% 3,3-diaminobenzidine plus 0.0033% H_2_O_2_ in 0.1 M Tris-HCl buffer (pH 7.4) for 5 min. The sections were then dehydrated, cleared and covered with neutral balsam. The slices were examined, and images were captured with an optical microscope equipped with a digital camera (Olympus). For double IF staining, the slices were incubated with a mixture of appropriate fluorescence-conjugated secondary antibodies for 2 h at room temperature and protected from light. After being thoroughly washed, nuclear chromatin was visualised using 4′,6-diamidino-2-phenylindole (DAPI). The sections were mounted with an anti-fading mounting medium and scanned using a confocal laser scanning microscope.

#### Sandwich enzyme-linked immunosorbent assay (ELISA)

The Aβ level in the APP/PS1 mouse brain was measured using human Aβ1–40 and human Aβ1–42 ELISA kits (Invitrogen) according to the manufacturer’s protocol. Briefly, the mouse cortex samples were lysed in ice-cold 20 mM Tris-HCl (pH 8.5) or 50 mM Tris-HCl containing 5 M guanidine HCl (pH 8.0) to analyse the contents of soluble Aβ and insoluble Aβ, respectively. The absorbance was measured at 450 nm using a 96-well plate reader.

IL-1β, IL-6 and TNFα secretion into the supernatants of the cultured cells was respectively measured with the commercial V-PLEX Human IL-1β kit, IL-6 ELISA kit and TNFα ELISA kit (Meso Scale Discovery), according to the manufacturer’s instructions. The absorbance was measured at a wavelength of 450 nm.

To measure DNA binding activity, nuclear extracts were obtained from the cultured cells using a nuclear extraction kit (Active Motif, Inc., Carlsbad, CA) according to the manufacturer’s instructions. The DNA binding activity of NFκB was examined with a TransAM ELISA kit (Active Motif).

#### Western blot assays

Protein was extracted from cells or tissue homogenates using RIPA buffer (pH 8.0) supplemented with a protease inhibitor cocktail. The protein concentration was measured using a BCA protein assay kit. Twenty-microgram of protein was loaded on sodium dodecyl sulfate polyacrylamide gels to separate the proteins. The separated proteins were then transferred onto polyvinylidene fluoride (PVDF) membranes (Millipore, Temecula, MA, USA), which were probed with the primary antibodies listed in Supplementary Table S1. After being rinsed, the PVDF membranes were incubated with appropriate HRP-conjugated secondary antibodies. The immunoreactive bands were visualised with an enhanced chemiluminescence kit (Pierce, Rockford, IL, USA), and the band density was quantified with Quantity One software. Blots were repeated at least three times for every condition.

#### Immunoprecipitation

To immunoprecipitate endogenous proteins extracted from cells or brain homogenates, 2 micrograms of protein was incubated with commercial primary antibodies against CEBPβ or control IgG for 16 h at 4 °C in a rotating incubator. The immune complexes were collected by incubation with True-Blot IP beads (eBioscience, Hatfield, United Kingdom) for another 2 h. The samples were rinsed with lysis buffer three times and eluted. Western blot analysis was performed with anti-NFκB antibodies.

#### Real-time PCR

Total RNA was extracted from brain tissues or cultured cells using an RNA isolation kit according to the manufacturer’s instructions. Two micrograms of total RNA from each sample was reverse transcribed using the Prime Script RT Reagent Kit. cDNA synthesis was performed at 37 °C for 15 min and 85 °C for 5 s. Quantitative real-time PCR was performed using SYBR Green PCR Master Mix on a 7300 Sequence Detection System. At least three independent analyses were conducted. The forward and reverse primers used for PCR are listed in Supplementary Table S2. The expression levels were calculated by normalising the cycle threshold (Ct) values to the total amount of β-actin. The expression levels in the treatment group are presented as a percentage of that in the control group.

#### Chromatin immunoprecipitation (ChIP)

ChIP was performed as previously described [34]. Mouse brain tissues were fixed with 4% paraformaldehyde for 12 h at 4 °C and neutralised with 0.125 M glycine for 10 min at 4 °C. After being washed twice with precooled PBS containing protease inhibitor cocktail, the samples were homogenised with 0.1% SDS lysis buffer containing 0.5% Triton X-100, 150 mM NaCl, 20 mM Tris-HCl (pH 8.0) and protease inhibitor cocktail. The samples were then sonicated and collected. One-third of the nuclear proteins were used as genomic DNA controls, and the remaining proteins were incubated with antibodies against NFκB for 18 h at 4 °C. Protein A-agarose beads were added to the reaction system and incubated with rotation for 1 h at 4 °C to capture the protein–DNA complexes. After being thoroughly rinsed, the specimens were collected by gentle centrifugation. The pellets were treated with elution buffer (pH 8.0), centrifuged and then treated with 5 M NaCl to reverse formaldehyde-induced crosslinking. After being heated for 12 h at 65 °C, the protein–DNA complexes were deproteinated with 20 mg/mL proteinase K for 1 h at 45 °C. The DNA samples were treated with phenol/chloroform and ethanol and amplified. Real-time PCR was carried out using primers for IL-6 and TNFα.

#### Lentiviral gene transfer of CEBPβ

A lentivirus vector expressing CEBPβ (LV-CEBPβ) was produced for gene transfer as previously described [35]. Briefly, all lentiviral vectors were generated by transient transfection into HEK293 cells. Forty-eight hours after transfection, the viruses were harvested, purified and concentrated by ultracentrifugation. The viral titres were measured, and a stock virus solution with a titre of 10^9^ infectious U/mL was generated. After preliminary dose-response assays, SH-SY5Y cells were plated in DMEM containing 10% FBS at a density of 2 × 10^5^ cells per cm^2^ and infected with 1000 microlitres of stock virus in 1.9 millilitres of the medium. After infection for 12 h at 37 °C, the SH-SY5Y cells were incubated with fresh DMEM without serum for 24 h.

#### Statistical analysis

One-way, two-way or repeated-measures analysis of variance (ANOVA) was used to evaluate differences as appropriate. The effects of genotype and treatment and the effect of the genotype × treatment interaction were analysed. In all cases, two-sided *P* values less than 0.05 were considered significant.

## Results

### Synthesis and characterization of the nanocarriers

**Fig. 1:**
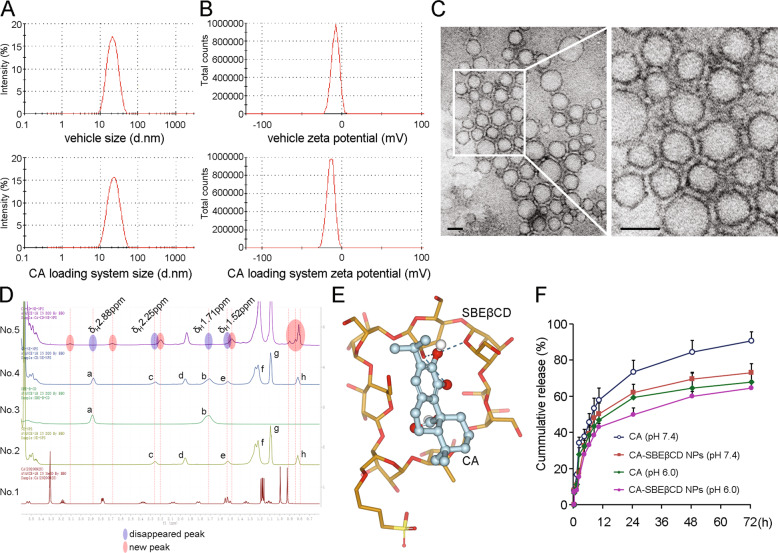
Preparation and characterization of CA-SBEβCD NPs. The size distribution (**A**) and zeta potential (**B**) of *SBEβCD NPs* and CA-*SBEβCD NPs* were determined by laser light scattering. **C** The morphology of *SBEβCD NPs* was assessed by negative staining with sodium phosphotungstate solution and examined by transmission electron microscopy. Scale bar: 50 nm. **D** ^1^H-NMR spectra of the components of CA-*SBEβCD NPs*. Resonance signals corresponding to the characteristic bonds of CA (No.1) appeared at 0.9–3.1 ppm. The characteristic peaks of SBEβCD (No.3) were at 2.88 and 1.71 ppm (peak a and b). NE (No.2), namely, nanoemulsions and auxiliary emulsion components without SBEβCD, presented signals at 2.55 ppm (peak c), 1.95 ppm (peak d), 1.52 ppm (peak e), 1.22 ppm (peak f), 1.09 ppm (peak g) and 0.83 ppm (peak h). The ^1^H-NMR spectrum of *SBEβCD NPs* (No.4) exhibited peaks in similar regions as SBEβCD and NE because the NPs contained SBEβCD and NE groups. Compared with CA*-SBEβCD NPs*, new peaks at chemical shifts of 0.80 to 1.00 ppm and 1.45 to 1.55 ppm appeared in the ^1^H-NMR spectrum of CA*-SBEβCD NPs* (No.5). Obvious increases in peak intensity at 1.15–1.35 ppm in the resonance signals of CA*-SBEβCD NPs* confirmed the synthesis of a link between CA and SBEβCD, and the signal peaks disappeared at 2.88 and 1.71 ppm suggests a bonding reaction between SBEβCD and CA. **E** Molecular docking diagram. CA potentially interacts with SBEβCD through hydrogen bonds: a hydroxyl group of CA respectively forms two intermolecular hydrogen bonds with the hydroxyl group and oxygen atom of SBEβCD; and a hydroxyl group of CA forms a hydrogen bond with a hydroxyl group of SBEβCD. The binding affinity was −21.35 kiloJoules/moL. **F** CA release from CA-*SBEβCD NPs* in PBS at pH 7.4 or 6.0 in vitro. CA dissolved in NE was used as control.

The internal cavity of SBEβCD is extended. The inclusion and dissolution rates of compound molecules of SBEβCD are higher than those of β-CD [36]. According to prior studies [33] and the EE and LE achieved in our study (Supplementary Table S3), SBEβCD and CA were prepared at a molar ratio of 1:1. Five percent (v/v) polyethylene glycol 400 (PEG 400) was used as the vehicle. The average particle sizes of the *SBEβCD NPs* and *CA-SBEβCD NPs* were 24.53 ± 5.14 nm and 36.42 ± 0.83 nm, respectively (Fig. 1A and Supplementary Table S3) and they had lower polydispersity indexes (PDIs) (0.243 ± 0.093 and 0.264 ± 0.004, respectively). The zeta potential of the *SBEβCD NPs* was −5.73 ± 2.12 mV, while that of the *CA-SBEβCD NPs* was −11.54 ± 1.45 mV (Fig. 1B and Supplementary Table S3). Transmission electron microscopy confirmed that the nanocarriers generally exhibited a regular spherical shape (Fig. 1C). The chemistry of the NPs was analysed by ^1^H-NMR spectroscopy indicating that CA was successfully introduced into the nanocarrier system (Fig. 1D). Molecular docking assays (Supplementary Method S1) suggest that CA interacts with SBEβCD *via* hydrogen bonds (Fig. 1E).

The release of CA from the nanocarriers was examined at pH 7.4 and pH 6.0 (37 °C). Continuous CA release from CA dissolved in NE and CA-*SBEβCD NPs* were observed in the first 4 h at pH 7.4 and pH 6.0. After 4 h, the release of CA gradually slowed. Compared with that of CA from CA-NE, the cumulative release rate of CA from CA-*SBEβCD NPs* was relatively lower (Fig. 1F). CA-*SBEβCD NPs* also maintained sustained release at pH 6.0, demonstrating the relative stability of the release behaviour.

### CA-*SBEβCD NPs* efficiently targets and enters the brain and is biologically safe according to preliminary studies

**Fig. 2:**
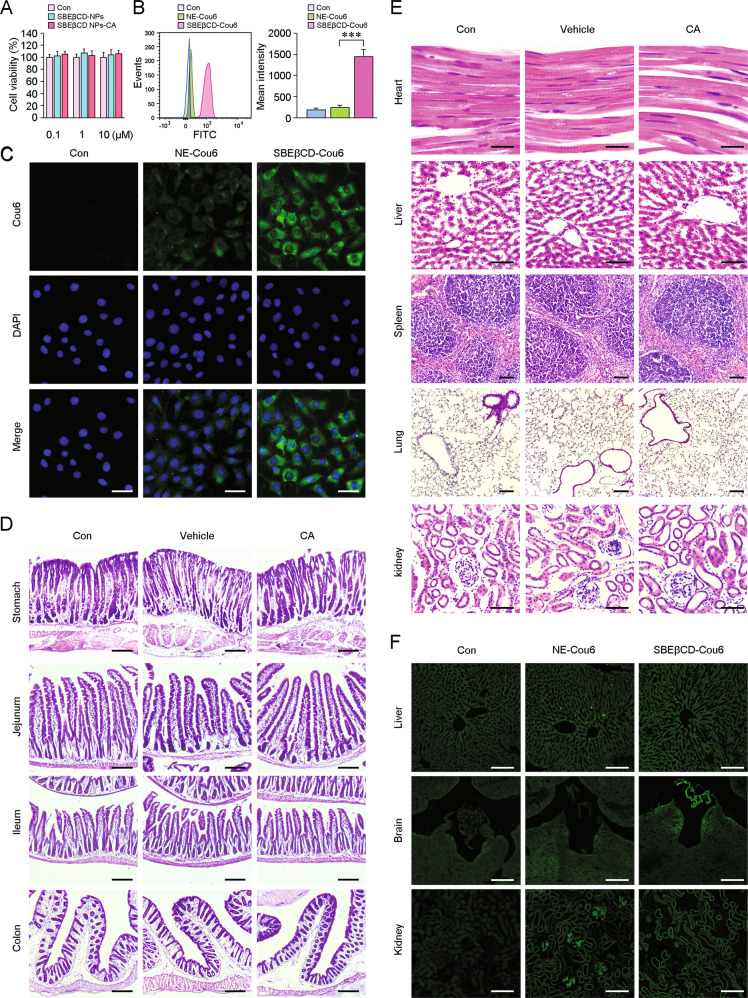
Evaluation of the transport efficiency and biological safety of CA-SBEβCD NPs. **A** In vitro cytotoxicity of *SBEβCD NPs*, NEs without SBEβCD and CA*-SBEβCD NPs*. SH-SY5Y cell viability was measured using a CCK-8 kit. Untreated cells were used as the controls (Con). **B** A Transwell assay in which hCMEC/D3 cells were plated in the upper chamber and SH-SY5Y cells were plated in the lower chamber was conducted to assess the ability of the cells to cross the BBB. The fluorescence intensity was determined by FCM to assess the levels of Cou6 in SH-SY5Y cells cocultured with hCMEC/D3 cells, which were treated with Cou6 suspensions, NE-dissolved Cou6 (NE-Cou6) or Cou6-loaded *SBEβCD NPs* (*SBEβCD*-Cou6). Cells treated by Cou6 suspensions in DMEM were used as the controls (Con). **C** Localisation analysis by laser scanning microscopy showed marked green Cou6 fluorescence in the cytoplasm of SH-SY5Y cells. Representative images showing the fluorescence signals of Cou6 in SH-SY5Y cells cocultured with hCMEC/D3 cells treated with different NP formulations. Scale bars: 20 μm. **D** H&E staining showing the morphology of stomach, jejunum, ileum and colon tissues from C57BL/6 mice orally gavaged with *SBEβCD NPs* or CA-*SBEβCD NPs* for 7 days. The tissue structure of the samples from each group was intact and the cells were neatly arranged. The connections between the cell layers were clear, and no obvious inflammatory cells were observed. Scale bars: 50 μm. **E** Safety assessment of the chronic administration with *SBEβCD NPs* or CA-*SBEβCD NPs* to mice for 30 days. Heart, liver, spleen, lung and kidney sections were stained with H&E. The structures of tissue were clear and intact, and the cells were normally arranged. No inflammatory cell infiltration was observed. Scale bars: heart, 5 μm; liver and kidney, 20 μm; spleen and lung, 50 μm. **F** Confocal laser scanning microscopy images showed the green fluorescence signals in the liver and kidney from the mice treated with NE-dissolved Cou6 and Cou6-loaded *SBEβCD NPs*. No fluorescence signals were observed in the brains of mice treated with NE-dissolved Cou6. Clear green Cou6 fluorescence were observed in the choroid plexus and periventricular region of the third ventricle from the brain sections of the mice administered with Cou6-loaded *SBEβCD NPs*. Scale bars: 50 μm. ***p < 0.001 vs the NE-Cou6 groups by one-way ANOVA. Values are presented as the means ± standard deviation (SD). All experiments were repeated at least three times.

The in vitro cytotoxic effects of the nanocarrier system were investigated using SH-SY5Y cells. There were no significant differences in the viability of cells treated with nanocarriers containing 0.1–10 µM CA and the viability of cells treated with other NPs (with or without equimolar concentrations of SBEβCD) (*p* > 0.05, Fig. 2A).

The efficiency of the nanocarriers in crossing the BBB was evaluated in hCMEC/D3 cells using the Transwell assay. As shown in Fig. 2B, higher fluorescence intensity was observed in SH-SY5Y cells cocultured with *SBEβCD NPs*-treated hCMEC/D3 cells in the upper chamber relative to that in SH-SY5Y cells cocultured with NE-Cou6-treated hCMEC/D3 cells (*p* < 0.001). Localisation analysis by laser scanning microscopy showed significant green Cou6 fluorescence in the cytoplasm of SH-SY5Y cells cocultured with hCMEC/D3 cells plus Cou6-*SBEβCD NPs*. The fluorescence was granular and scattered (Fig. 2C).

To assess acute toxicity, stomach, jejunum, ileum and colon samples from each mouse orally gavaged with *SBEβCD NPs* (vehicle) or *CA-SBEβCD NPs* (CA) every day for 7 days were subjected to H&E staining. No, obviously abnormal status was observed among the groups (Fig. 2D). For chronic toxicity, a 30 days-administration above were performed. H&E staining showed no marked organ damage in the heart, liver, spleen, lung and kidney (Fig. 2E). To verify the transport efficiency of the nanocarriers in vivo, the fluorescence signals in brain sections were assessed by confocal laser scanning microscopy after oral administration of Cou6-labelled NPs (Fig. 2F).

### CA-*SBEβCD NPs* treatment alleviates cognitive impairment in APP/PS1 mice

**Fig. 3:**
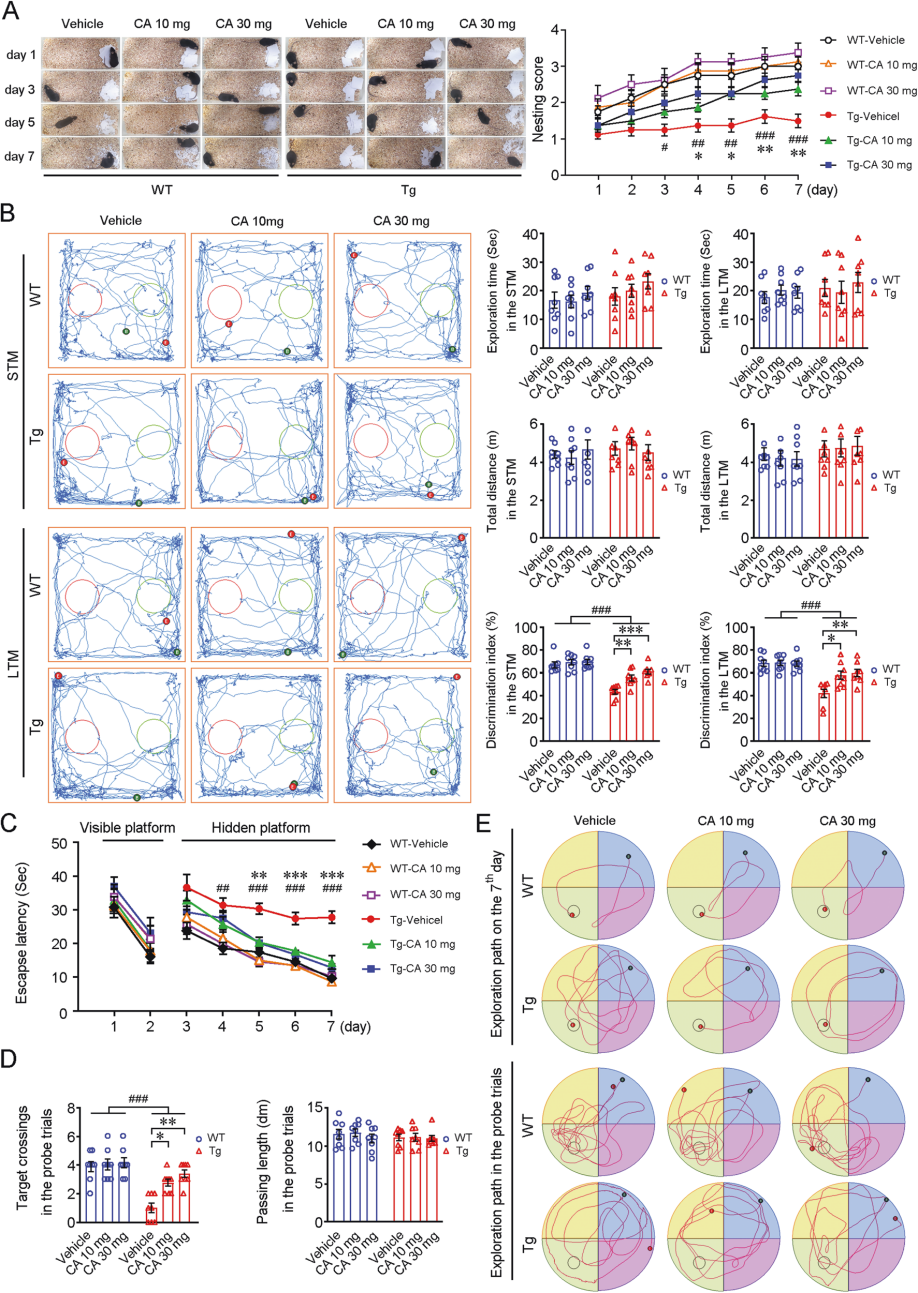
Evaluation of the effect of CA-SBEβCD NPs administration on the learning and memory of APP/PS1 mice. Four-month-old APP/PS1 (Tg) mice and age-matched C57BL/5 (WT) mice were orally gavaged with *SBEβCD NPs* (vehicle) or *SBEβCD NPs* loaded with 10 or 30 mg/kg CA for 5 months. **A** Representative images showing nests constructed by the mice. Nesting scores were quantified. **B** The recognition memory of WT and Tg mice was assessed by the NORT. The traces of the mice in the STM and LTM tests were recorded. The green dots and red dots respectively represent the starting and ending locations of the mice. Tg mice and WT mice showed similar total time and distance during the habituation phase. The discrimination index was used to compare the ability of the mice to recognise the familiar object. **C** Spatial learning and memory were evaluated by the MWM test. WT and Tg mice exhibited comparable escape latencies during the visible platform training phase. Tg mice performed longer escape latencies than WT mice in the hidden platform test from the 3rd day: CA treatment shortened the escape latency of Tg mice. **D** On the final day (the 8th day), the platform was removed, and the probe trial was performed. The number of times and the navigated distance of each mouse crossed the area in which the platform was previously located were quantified. **E** Representative traces show the exploration of the mice on the 7th and 8th day. *p < 0.05, ** *p* < 0.01, ***p < 0.001 vs vehicle-treated mice; ^#^*p* < 0.05, ^##^*p* < 0.01 and ^###^*p* < 0.001 vs the WT mice by the repeated-measures ANOVA. Values represent the mean ± standard error of the mean (SEM). *n* = 8 in each group.

CA-*SBEβCD NPs* were orally administered to transgenic APP/PS1 mice (Tg) and age-matched wild-type (WT) C57BL/6 mice at a dose of 10 or 30 mg/kg beginning at 4 months of age. After 5 months of treatment, cognition was assessed. As shown in Fig. 3A, Tg mice exhibited deficits in nesting behaviour compared with the WT mice. The nesting scores of the CA-*SBEβCD NPs* (30 mg/kg CA)-treated Tg group were higher than those of *SBEβCD NPs-*treated Tg mice (vehicle controls) from the 4th day to the 7th day (Fig. 3A). The Tg mice presented poor recognition memory in the NORT (Fig. 3B). The discrimination index CA-*SBEβCD NPs*-treated Tg mice were higher than vehicle-treated Tg mice in the STM (F(2,21) = 16.516, *p* < 0.001) and LTM (F(2,21) = 7.487, *p* < 0.01) test. CA-*SBEβCD NPs* treatment markedly improved the spatial learning ability of the Tg mice by shortening the escape latency during the hidden platform test (Fig. 3C, E) and increasing the frequency without altering total distances during the probe trial (Fig. 3D, E) in the in MWM test.

### CA-*SBEβCD NPs* treatment mitigates AD-like pathology in the brains of APP/PS1 mice

**Fig. 4:**
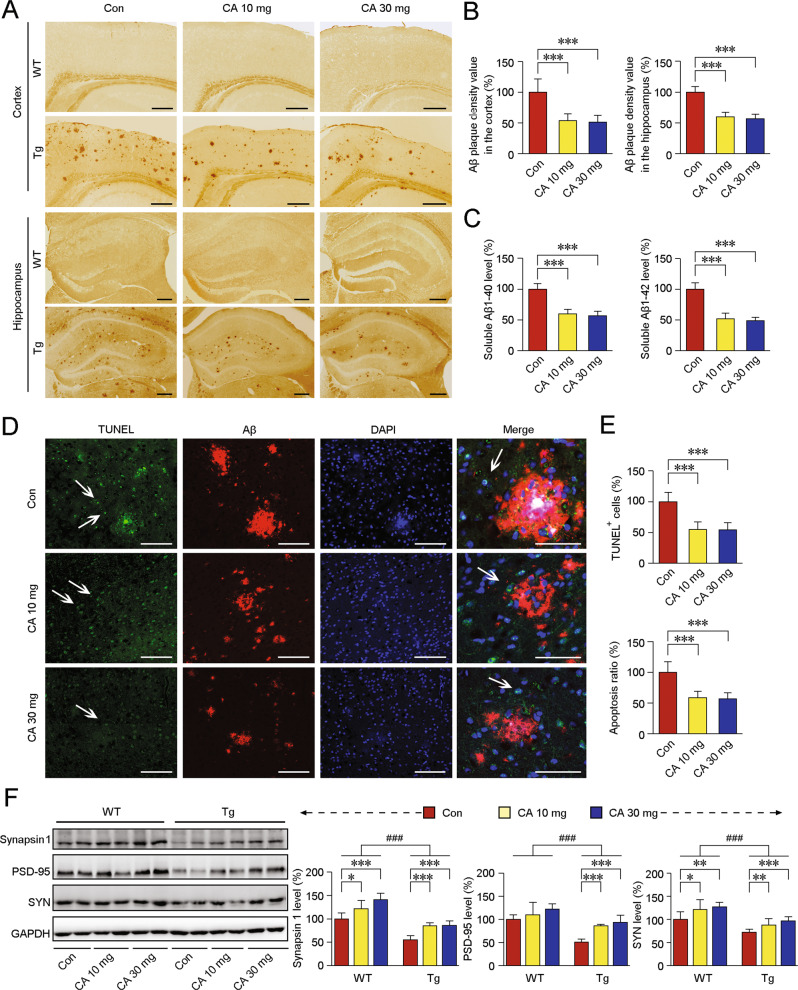
Effects of CA-SBEβCD NPs treatment on AD-related pathology in the APP/PS1 mouse brain. **A** Aβ-positive senile plaques were detected in APP/PS1 (Tg) mice and age-matched C57BL/6 (WT) mice treated with *CA-SBEβCD NPs* (loaded with 10 or 30 mg/kg of CA) or *SBEβCD NPs* (vehicle control) for 5 months. **B** The Aβ plaque density in the cerebral cortex and hippocampus was quantified. **C** Soluble Aβ40 and Aβ42 levels in the cortex were measured by ELISA. **D** Confocal laser scanning microscopy images showing apoptosis around Aβ-positive areas. TUNEL staining (green) showing apoptotic cells around Aβ plaques (red). DAPI was used to label the nuclei (blue). Scale bars: 100 μm. The white arrows indicate apoptotic cells. The high-magnification images in the right panels show the localisation of apoptotic cells around the Aβ-positive deposits. Scale bar = 60 μm. **E** Quantification of the apoptotic cells. **F** Western blot analysis showing the expression of the synaptic proteins, synapsin 1, PSD-95 and SYN. Blots were repeated at least three times for every condition.*p < 0.05, **p < 0.01 and ***p < 0.001 vs vehicle-treated mice by the one-way ANOVA (**A**–**E**). *p < 0.05, **p < 0.01, ***p < 0.001 vs vehicle-treated mice; ^###^*p* < 0.001 vs the WT mice by the two-way ANOVA with post hoc Fisher’s least significant difference (LSD) tests. Values represent the mean ± SD. *n* = 8 in each group.

Immunohistochemical staining showed that fewer Aβ plaques were observed in the CA-treated Tg mice than in those of vehicle-treated Tg mice (*ps* < 0.001, Fig. 4A, B). Furthermore, ELISA assays showed that the levels of soluble Aβ42 and Aβ40 were significantly reduced after CA treatment (*ps* < 0.001, Fig. 4C). TdT-mediated dUTP nick end labelling (TUNEL) staining showed that apoptosis cells were less in the brains of CA-treated Tg mice than in those of vehicle*-*treated Tg mice (Fig. 4D, E). We investigated the protein levels of synaptophysin (SYN, a presynaptic vesicle protein), synapsin 1 (a synaptic vesicle-related protein) and PSD-95 (a postsynaptic density protein) by immunoblotting. As shown in Fig. 4F, CA treatment alleviated the decline of these protein levels (*ps* < 0.001) in the Tg mice. CA treatment also affected the protein expression of SYN (F(2,21) = 5.547, *ps* < 0.05) and synapsin 1 (F(2,21) = 12.847, *ps* < 0.001) in the hippocampi of the WT mice.

### CA-*SBEβCD NPs* administration alleviates the abnormal aggregation of glia in the brains of APP/PS1 mice

**Fig. 5:**
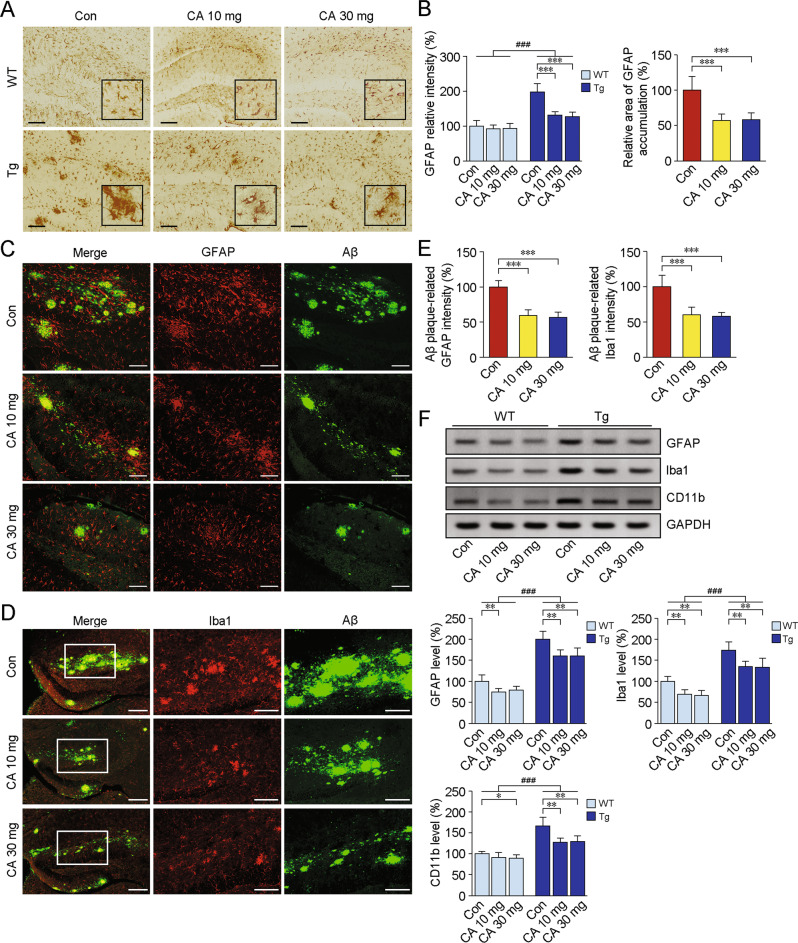
Effects of CA-SBEβCD NPs treatment on microglial and astrocyte activation in the brains of APP/PS1 mice. APP/PS1 Tg mice and age-matched WT C57BL/6 mice were administered with *CA-SBEβCD NPs* (loaded with 10 or 30 mg/kg CA) or *SBEβCD NPs* (vehicle control) for 5 months. **A** Astrocytes in the hippocampus were detected by IHC with an anti-GFAP antibody. In the brains of Tg mice, astrocytes aggregated and exhibited somatic hypertrophy and thickening of the primary processes. Scale bars: 200 μm. **B** Quantification of the GFAP intensity in WT and Tg mice. **C** Representative confocal laser scanning microscopy images showing the distribution and localisation of activated astrocytes and Aβ plaques in the hippocampus. GFAP-labelled astrocytes (red) around Aβ plaques (green) are shown. Scale bars: 100 μm. **D** Double labelling for Iba1 (red) and Aβ (green) respectively shows the activated microglia and Aβ plaques in the hippocampus of the Tg mouse. Scale bars: 200 μm. The high-magnification images in the right panels correspond to the areas indicated in the boxes. Scale bars: 100 μm. **E** Quantification of the relative intensities of GFAP and Iba1. **F** The protein levels of GFAP, Iba1 and CD11b were examined by Western blot analysis. Blots were repeated at least three times for every condition. *p < 0.05, **p < 0.01 and ***p < 0.001 vs vehicle-treated mice; ^###^*p* < 0.001 vs the WT mice by the one- or two-way ANOVA with post hoc Fisher’s LSD tests as appropriate. Values represent the mean ± SD. *n* = 6 in each group.

Considering the anti-inflammatory properties of CA and the deleteriousness of inflammation in promoting aberrant synaptic pruning and facilitating Aβ pathology [37], we investigated the effects of CA-*SBEβCD NPs* treatment against neuroinflammation. As shown in Fig. 5A, B, the immunostaining signals of GFAP, an astrocyte marker, were increased in the brains of Tg mice than those of WT mice (F(1,30) = 274.485, *p* < 0.001). CA-*SBEβCD NPs* treatment alleviated the accumulation of astrocytes in the Tg mice brains. The intensity of GFAP around Aβ plaques in the CA-treated Tg mice was reduced compared with that in the vehicle-treated Tg mice (Fig. 5C, E). Moreover, the intensity of Iba1 immunoreactivity around Aβ plaques was markedly attenuated under CA treatment in the Tg mice brains (Fig. 5D, E). The protein expression of GFAP, Iba1 and CD11b (a microglial marker) was determined by Western blot analysis, and the observed changes in expression were consistent with above-mentioned changes caused by CA-*SBEβCD NPs* treatment (Fig. 5F).

### CA-*SBEβCD NPs* administration reduces the release of IL-1β, IL-6 and TNFα and diminishes the interaction of CEBPβ with NFκB in the APP/PS1 mouse brain

**Fig. 6:**
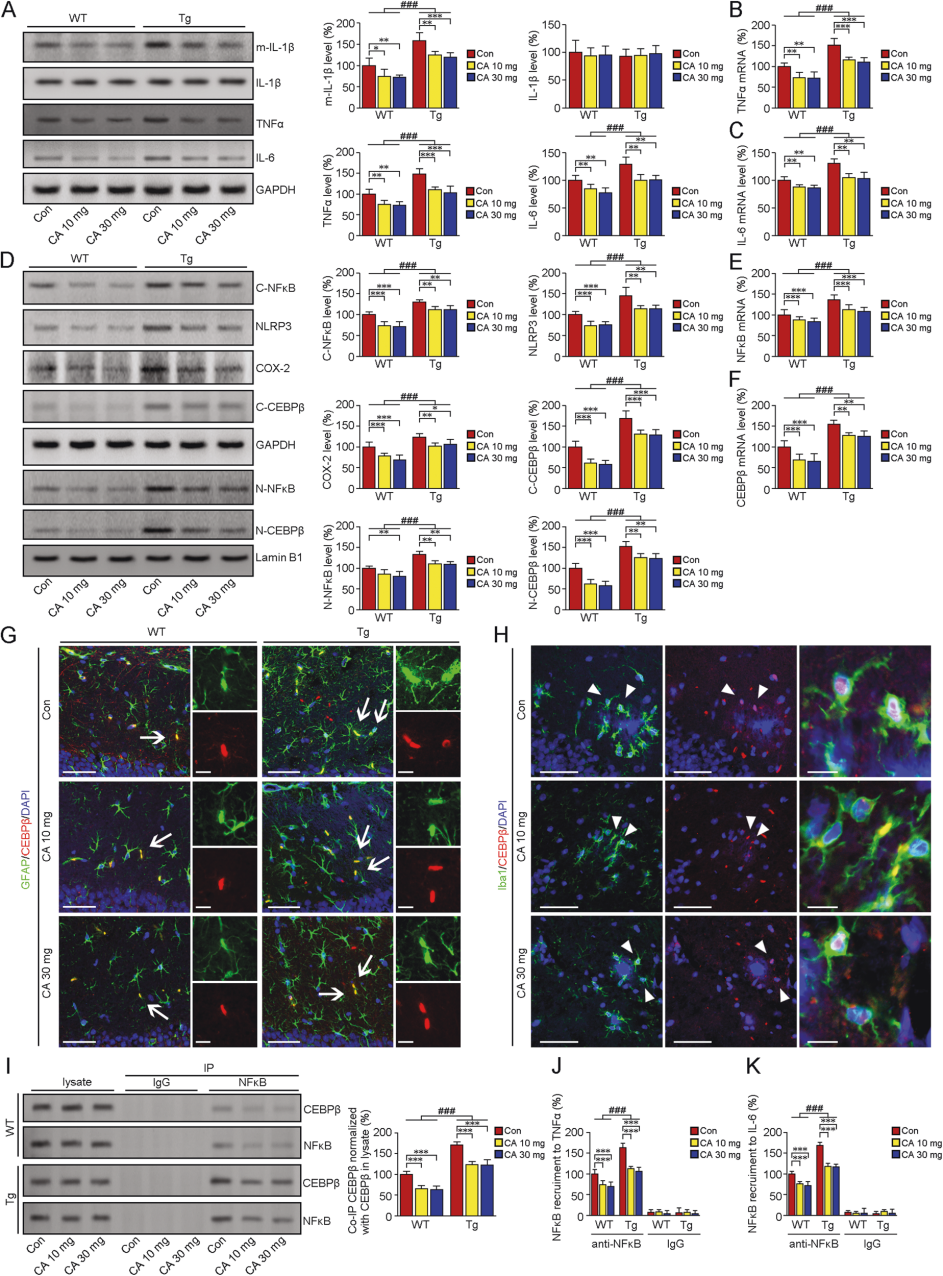
CA-SBEβCD NPs-mediated inhibition of proinflammatory cytokines is involved in the regulation of CEBPβ in the brains of APP/PS1 mice. *CA-SBEβCD NPs* (loaded with 10 or 30 mg/kg CA) or *SBEβCD NPs* (vehicle control) were administered to 4-month-old APP/PS1 Tg mice and age-matched WT C57BL/6 mice for 5 months. **A** Western blot and quantitative analysis showing the protein levels of pro- and mature IL-1β, IL-6 and TNFα in the mouse brain. Real-time PCR analysis showing the mRNA levels of TNFα (**B**) and IL-6 (**C**). **D** Western blot analysis of NFκB p65 expression in the cytosol and nucleus in protein lysates from Tg and WT mice brains. The protein expression levels of COX-2 and NLRP3 were analysed. Representative blots showing the protein levels of cytosolic and nuclear CEBPβ. The mRNA levels of NFκB (**E**) and CEBPβ (**F**). **G** Representative images of immunofluorescence (IF) staining for CEBPβ (red) and GFAP (green) in the hippocampi of WT and Tg mice. The nuclei were labelled with DAPI (blue). The white arrows indicate colocalization of CEBPβ and GFAP. Scale bars: 60 µm. The enlarged images in the right panels show GFAP- and CEBPβ-positive cells. Scale bars: 10 µm. **H** IF labelling of CEBPβ and Iba1 in the hippocampus of Tg mice. Scale bars: 60 µm. The white arrowheads indicate the colocalizations of CEBPβ and Iba1. Right panels: enlarged images of the indicated cells. Scale bars: 10 µm. **I** The interaction of CEBPβ with NFκB was assessed by Co-IP. ChIP was performed to assess the NFκB-mediated transcription of IL-6 (**J**) and TNFα (**K**). *p < 0.05, **p < 0.01 and ***p < 0.001 vs vehicle-treated mice; ^###^*p* < 0.001 vs the WT mice by the two-way ANOVA with post hoc Fisher’s LSD tests. Values represent the mean ± SD. *n* = 6 in each group. Blots were repeated at least three times for every condition.

Neuroinflammation, even in the earliest stages, induces the release of proinflammatory factors and leads to neuronal injury [17]. As shown in Fig. 6A, the protein levels of mature IL-1β (F(1,30) = 101.522, *p* < 0.001), TNFα (F(1,30) = 87.968, *p* < 0.001) and IL-6 (F(1,30) = 40.036, *p* < 0.001) were increased in the brains of Tg mice than those of WT mice. CA-*SBEβCD NPs* treatment diminished the protein levels above in the brains of both genotypes without altering pro-IL-1β protein levels (*ps* > 0.05). The mRNA levels of TNFα (F(1,30) = 39.083, *p* < 0.001, Fig. 6B) and IL-6 (F(1,30) = 40.036, *p* < 0.001, Fig. 6C) were higher in the brains of Tg mice than those of WT mice. CA-*SBEβCD NPs* treatment suppressed the gene expression of TNFα and IL-6 in both genotypes (*ps* < 0.001).

To elucidate the effects of CA on regulating inflammatory signals, we analysed the expression of NFκB. CA management relieved the increases of protein levels in both nuclear- and cytosolic NFκB p65 subunit in the Tg mice brains (Fig. 6D, *ps* < 0.001). The effects of CA treatment on NFκB mRNA levels were as well (*ps* < 0.001, Fig. 6E). We examined the expression of COX-2, a downstream target of NFκB involved in the inflammatory process. The elevations of COX-2 protein expressions in the Tg mice brains were suppressed by CA-*SBEβCD NPs* treatment (*ps* < 0.01). It has been reported that IL-1β-related NLRP3 expression is involved in NFκB activation [38]. In the present study. The increases of NLRP3 protein expression in the Tg mice brains were mitigated under CA-*SBEβCD NPs* treatment. CEBPβ is a critical regulator of IL-6 production [26], and CEBPβ is essential for regulating IL-1β and TNFα secretion [39]. CEBPβ acts upstream of NFκB p65, modulating the maturation of IL-1β [20]. The interactions of NFκB with CEBPβ enhance the transcriptional activity of NFκB [24]. In our study, CA-*SBEβCD NPs* treatment alleviated the elevation in both CEBPβ protein expressions (Fig. 6D) and CEBPβ mRNA levels (Fig. 6F) in the Tg mice. IF double labelling of CEBPβ with GFAP (Fig. 6G) or Iba1 (Fig. 6H) showed the CEBPβ expression and its relationship with astrocytes and microglia in the hippocampi of the mice.

To investigate whether the CA-induced decrease in CEBPβ expression could modulate the interaction of CEBPβ with NFκB, we examined the CEBPβ-NFκB p65 complex by coimmunoprecipitation (Co-IP). As shown in Fig. 6I, CA treatment relieved the increases of CEBPβ-NFκB p65 interactions in the Tg mice brains. Meanwhile, ChIP assays showed that CA administration mitigated the elevation of NFκB recruitment to TNFα (Fig. 6J) and IL-6 (Fig. Fig. 6K) in the Tg group (*ps* < 0.001).

### CA provides neuroprotection and attenuates neuroinflammation by regulating CEBPβ/NFκB

**Fig. 7:**
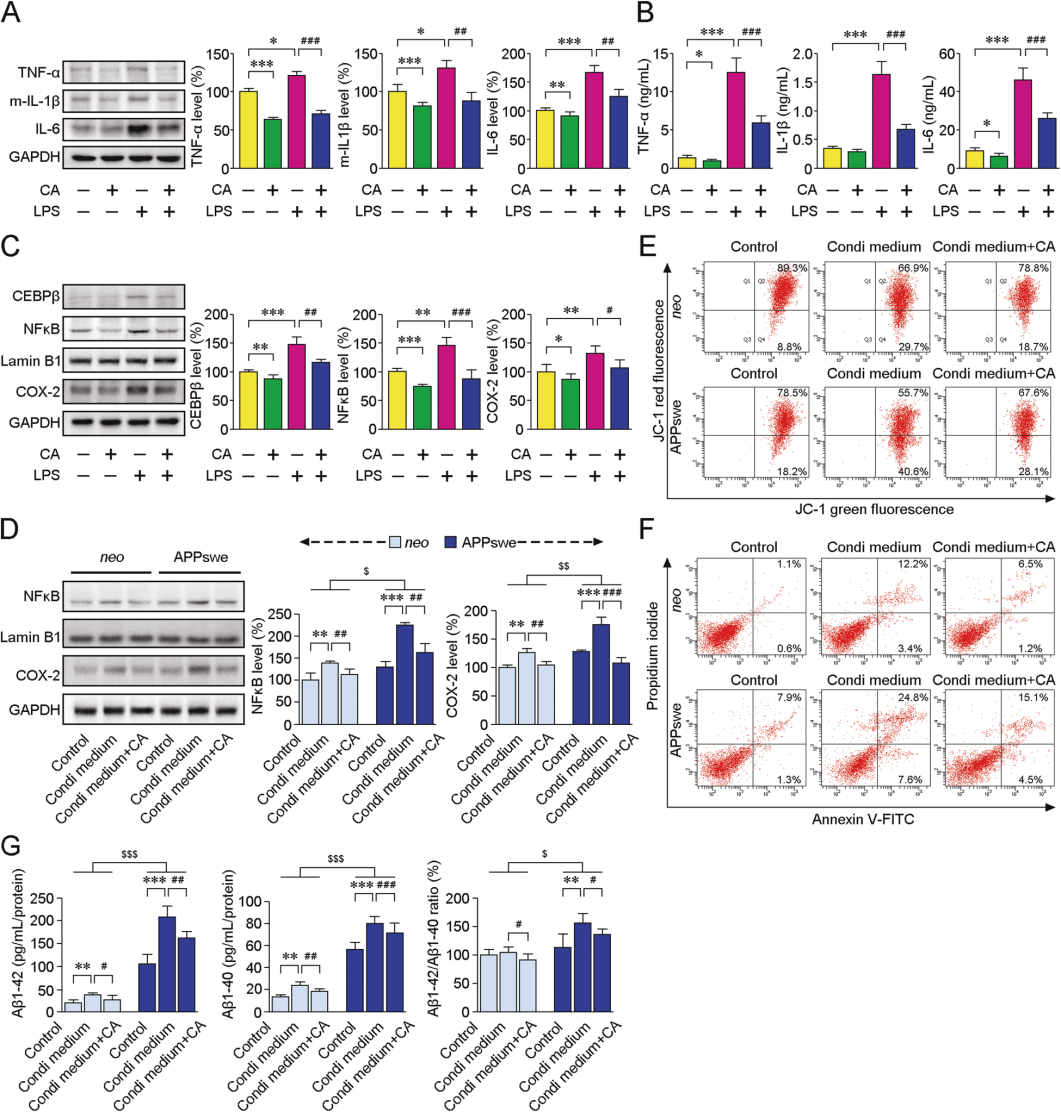
CA treatment abolishes the effects of glia-secreted proinflammatory cytokines on neuronal damage and Aβ secretion. A172 human glioblastoma cells were incubated with 1 µg/mL LPS for 24 h to trigger proinflammatory responses and then treated with CA at a final concentration of 1 µM for 12 h. **A** Western blot analysis was performed to evaluate the protein expression of IL-1β, IL-6 and TNFα. **B** The secretion of IL-1β, IL-6 and TNFα was measured by ELISA. **C** Representative blots showing the protein levels of COX-2, nuclear CEBPβ and NFκB in A172 cells. **D** SH-SY5Y cells overexpressing human APPswe or empty vector (*neo*) were cultured in LPS-primed cell-conditioned medium and treated with or without CA. The protein levels of nuclear NFκB and COX-2 were measured. **E** The mitochondrial transmembrane potential of APPswe and *neo* cells was determined by FCM. The percent of red and green fluorescence signal of JC-1 was measured to assess the degree of mitochondrial depolarisation. **F** Apoptosis of APPswe and *neo* cells were labelled by Annexin V-FITC/PI staining and analysed by FCM. **G** The secretion of Aβ40 and Aβ42 by APPswe and *neo* cells was measured by ELISA. The ratio of Aβ42 to Aβ40 was quantified. The data were representative of at least three independent experiments. *p < 0.05, **p < 0.01 and ***p < 0.001 vs untreated cells; ^#^*p* < 0.05, ^##^*p* < 0.01 and ^###^*p* < 0.001 vs the LPS-stimulated group (**A**–**C**). **p < 0.01 and ***p < 0.001 vs the group treated with the medium of A172 cells not incubated with LPS (Control); ^#^*p* < 0.05, ^##^*p* < 0.01 and ^###^*p* < 0.001 vs the group incubated with the medium of LPS-primed A172 cells without CA treatment; ^$^*p* < 0.05, ^$$^*p* < 0.01 and ^$$$^*p* < 0.001 vs *neo* cells (**D**, **G**). Data were submitted to two-way ANOVA with Fisher LSD post hoc tests. Values represent the mean ± SD. All experiments were repeated at least three times.

It has been demonstrated that silencing CEBPβ alleviates glial activation and reduces dopaminergic damage in a Parkinson’s disease model [23]. Based on some of the common molecular mechanisms of neurodegeneration, we investigated whether CA-induced CEBPβ inhibition is related to modulations of the glial cascade and exerts neuroprotective effects in an AD cell model in vitro. As shown in Fig. 7A, the LPS-triggered elevations of TNFα (*p* < 0.001), mature IL-1β (*p* < 0.01) and IL-6 (*p* < 0.01) protein levels were attenuated by CA treatment, and the effects of CA on the IL-1β, IL-6 and TNFα secretions in the medium of LPS-primed A172 cells were as well (*ps* < 0.001, Fig. 7B). CA treatment mitigated LPS-induced increases of CEBPβ (*p* < 0.01) and NFκB (*p* < 0.001) in the nuclei, and COX-2 protein expressions in the cytoplasm (*p* < 0.05) (Fig. 7C). Meanwhile, CA treatment diminished the upregulation of nuclear NFκB and COX-2 expressions (Fig. 7D), alleviated mitochondrial lesions (Fig. 7E) and diminished apoptosis (Fig. 7F) in the *neo* and APPswe cells incubated with LPS-primed cell-condition medium. CA treatment also mitigated the increases of Aβ40 and Aβ42 secretions in *neo* and APPswe cells treated with the conditioned medium (Fig. 7G).

**Fig. 8:**
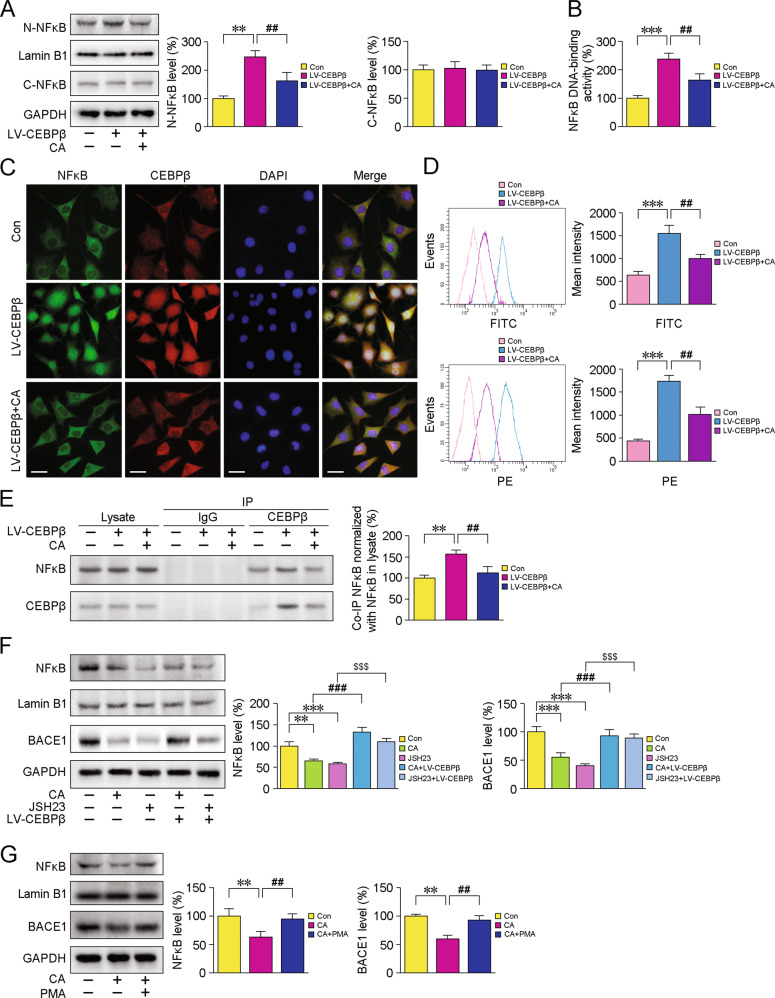
CA-mediated inhibition of CEBPβ/NFκB signalling directly inhibits BACE1. **A** Representative blots of NFκB p65 in SH-SY5Y cells following transfection with a lentiviral vector-encoding human CEBPβ (LV-CEBPβ) or treatment with LV-CEBPβ plus CA. **B** The DNA binding activity of NFκB was determined by ELISA. **C** IF staining shows the NFκB p65 (green) and CEBPβ (red) protein expressions. Scale bars: 20 µm. **D** FCM analysis of NFκB p65 (FITC channel) and CEBPβ (PE channel) after treatment with LV-CEBPβ or LV-CEBPβ plus CA. **E** The binding of CEBPβ to NFκB was examined by Co-IP. **F** Similar to JSH-23, CA treatment-induced decreases in nuclear NFκB p65 and cytosolic BACE1 expressions were blocked by LV-CEBPβ. **G** CA-mediated inhibition of BACE1 was diminished by the NFκB activator, PMA. The data were representative of at least three independent experiments. **p < 0.01 and ***p < 0.001 vs the controls; ^##^*p* < 0.01 vs the LV-CEBPβ-treated group (**A**–**E**). **p < 0.01 and ***p < 0.001 vs the controls; ^###^*p* < 0.001 versus CA-treated group; ^$$$^*p* < 0.001 versus JSH-23 treatment group (**F**). **p < 0.01 vs the controls; ^##^*p* < 0.01 vs the CA treatment group (**G**). Data were analysed using one-way ANOVA and Fisher LSD post hoc tests. Values represent the mean ± SD of at least three independent experiments.

Interactions of NFκB with BACE1 enhanced the transactivation of BACE1 [40], facilitating the amyloidogenic pathway. Considering the role of CEBPβ as the upstream regulator of NFκB [20], we investigated the specificity of CA for suppressing the transcriptional activity of NFκB and verified that CA downregulated BACE1 expression via the CEBPβ/NFκB signalling pathway. We delivered CEBPβ to SH-SY5Y cells using a lentiviral vector-encoding human CEBPβ (LV-CEBPβ). As shown in Fig. 8A, CA treatment mitigated the LV-CEBPβ pretreatment-caused increases of nuclear NFκB p65 protein levels in the SH-SY5Y cells (*p* < 0.01). CA addition ameliorated LV-CEBPβ-triggered increases of NFκB DNA binding activity (Fig. 8B, *p* < 0.01). IF staining of NFκB p65 confirmed those results above (Fig. 8C). FCM analyses showed that LV-CEBPβ-treated cells exhibited the highest intracellular fluorescence intensity for NFκB p65 and CEBPβ (*ps* < 0.001). By contrast, CA addition diminished LV-CEBPβ-caused increases of NFκB p65 and CEBPβ expressions (Fig. 8D, *ps* < 0.01). Co-IP assays indicated that CA treatment relieved LV-CEBPβ-induced elevation of CEBPβ-NFκB interactions (*p* < 0.01, Fig. 8E). The protein levels of nuclear NFκB p65 and BACE1 were reduced after treatment with CA or an NFκB activation inhibitor, JSH-23 (20 µM for 6 h); however, these effects were diminished by LV-CEBPβ treatment (*ps* < 0.001, Fig. 8F). Furthermore, we observed that administration of an NFκB activator, phorbol myristate acetate (PMA, 30 ng/mL for 6 h), blocked the CA-induced decreases in BACE1 and NFκB protein expressions (*ps* < 0.01, Fig. 8G).

## Discussion

Inflammatory alterations occur prior to the appearance of Aβ plaques [41]. During 'inflammageing', cytokine network dysfunction is associated with ageing and age-related diseases [42]. As critical components of the innate immune system in the central nervous system, microglia and astrocytes, produce cytokines and perform monitoring [43]. IL-1β secretion at physiological doses by microglia is required for long-term potentiation (LTP) induction, learning and memory formation [44]. A low dose of TNFα can regulate synaptic function by modulating AMPA receptor levels, synaptic strength and homeostatic plasticity [45]. However, ageing is related to increased and persistent inflammation [42]. Astrocytes and microglia take on opposite roles with age, as they increase the production of proinflammatory cytokines and reduce the production of anti-inflammatory cytokines [46]. Chronic inflammation is a common occurrence in ageing and AD [47]. A sustained inflammatory response is observed in the brains of AD patients [3]. Sustained activation of microglia weakens their capacity to bind and phagocytise Aβ and decreases Aβ degradation [48]. However, microglia still produce proinflammatory cytokines [49]. Importantly, a high level of IL-1β impairs LTP and hinders learning and memory in a dose-dependent manner [44].

NFκB signalling pathway plays important role in cellular responses to neuronal injury and synaptic plasticity in the central nervous system [50]. NFκB modulates immunity by regulating the transcription of cytokines and immune response genes. In response to stimuli, NFκB translocates from the cytosol to the nucleus to induce the transcription of target genes [51]. Importantly, NFκB signalling is directly associated with spinogenesis and strengthens synapse connections during learning and memory formation [52]; however, NFκB activation in neurons of the aged brain was shown to be incredibly toxic [53]. Sustained activation of NFκB is consistent with the chronic inflammation observed in areas of the brain undergoing atrophy [54]. Activated NFκB is clearly observed in neurodegenerating cells of postmortem brains of AD patients [55]. Aβ neurotoxicity is dependent on NFκB during gliosis [56]. In contrast, blocking NFκB lessened IL-1β-induced degeneration [57]. Disrupting the binding of NFκB to the promoter of *Bace1* suppresses inflammatory processes [58]. NFκB inhibition is considered a potential target for reducing neuroinflammatory damage and thus ameliorating AD.

**Fig. 9:**
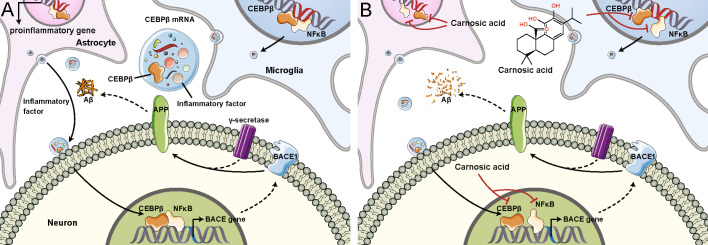
Schematic diagram of CA on the regulation of CEBPβ–NFκB interaction in the AD brain. **A** The increases of CEBPβ–NFκB interaction enhance NFκB DNA binding activity, facilitating the transcription of NFκB target genes. The upregulation of CEBPβ and NFκB downstream genes, proinflammatory cytokines, could increase the releases of inflammatory factor from the microbubbles or exosomes secreted by microglia or astrocytes. NFκB-mediated increases and activation of BACE1 prompt the amyloidogenic processing of APP, followed by the γ-secretase cleavage, leading to the Aβ production by the neuron. Chronic inflammatory conditions and Aβ depositions exacerbate the binding of CEBPβ-NFκB, deteriorating the AD-like pathology. **B** CA provides neuroprotective effects by preventing the nucleus translocation of NFκB, reducing the expressions of proinflammatory cytokines. Meanwhile, CA reduces the CEBPβ–NFκB interaction and alleviates Aβ aggregation via inhibiting the amyloidogenic proteolytic pathway of APP.

Some natural products, such as polyphenols [59] and alkaloids [60], can alleviate AD-related lesions by inhibiting NFκB signalling pathways. In vivo experiments showed that CA can suppress NFκB activation and alleviate high fat diet-induced brain injury in mice [61]. Interestingly, CA is considered a neuroprotective agent due to its anti-inflammatory and mitochondrial protective effects [62]. In our study, CA treatment alleviated the elevation of protein expression of nuclear NFκB p65 in the Tg mice brains. Similarly, CA treatment downregulated the protein and mRNA levels of the inflammatory biomarkers IL-6, TNFα and COX-2, suggesting that CA-induced inhibition of NFκB nuclear translocation suppresses downstream inflammation-related signals. Moreover, CA treatment significantly reduced LPS-primed cell apoptosis and diminished Aβ42 and Aβ40 secretion in vitro; these findings are similar to those of a previous study [63]. The relationship between CEBPβ and NFκB initially received attention because of its regulatory effects on IL-6 [64] and IL-1β [20]. In CEBPβ-null mice, the expression of proinflammatory genes and the neurotoxicity caused by activated microglia are mitigated [22]. CEBPβ protein expression is elevated in the cortex of the brains from AD patients and the aged brain [25]. Wang and colleagues reported that CEBPβ was significantly increased in a 3×Tg-AD mouse model, which harboured the variants of the genes, Psen1 allele, APP Swedish mutation and MAPT allele. Aβ-plaque depositions and hyperphosphorylated tau are observed in the hippocampus of the mouse model between 12–15 months of age. Knockout of CEBPβ in this 3×Tg-AD mice could reduce above AD-like pathologies *via* inhibiting δ-secretase [65]. Interestingly, CEBPβ overexpression induced by hippocampal stereotactic injection with Adeno-associated virus vector-encoding CEBPβ led to the upregulation of ApoE4 in this 3×Tg-AD mice and caused the losses of dendritic spines in the hippocampal neurons [66]. These results indicate the significance of CEBPβ as a pivotal transcription factor for the genes on regulating APP processing and tau pathology in AD. Further animal experiments need to be performed to address the effects of CA-*SBEβCD NPs*-induced CEBPβ inhibition on its other downstream targets. Knocking down CEBPβ reduces NFκB p65 subunit expression and inhibits NFκB p65 activation, whereas silencing p65 does not affect CEBPβ [20]. The interaction of NFκB with CEBP through the bZIP region can enhance the transcriptional activity of NFκB [24]. Upregulating CEBPβ expression enhanced the binding activity of p65 to the promoter of IL-1β; accordingly, inhibition of CEBPβ suppressed the DNA binding activity of NFκB [20]. We propose that suppressing the abnormal increase in CEBPβ expression and inhibiting the interaction of CEBPβ with NFκB might disturb the proinflammatory environment and block AD progression. In our study, we showed that CA administration ameliorated the aberrant increase in CEBPβ protein expression in the Tg mice brains. CA reversed the inflammatory mediator-induced activation of CEBPβ/NFκB signalling in vitro and reduced Aβ secretions *via* inhibiting BACE1. These findings provide a plausible link between targeting proinflammatory cytokine transcription and inhibiting amyloidogenesis. Interestingly, our molecular docking result suggests that CA interacts with CEBPβ through hydrogen bonds. The amino acid residues of CEBPβ forming hydrogen bonds with CA are Asparagine-296 and Alanine-292. The binding affinity was −25.12 KJoule/moL (Supplementary Fig. S1). The results of molecular docking indicate the potential of CA on CEBPβ intervention. Further studies of the postmortem brains of AD patients may help elucidate the exact effects of CEBPβ on regulating NFκB target genes during AD pathology (Fig. 9). Our results in combination with previous findings regarding the neuroprotective effects of CA [63] confirmed that CA has therapeutic potential for AD. Limitations of this study include that the molecular docking only mimics the interactions of CA and CEBPβ; the CEBPβ immunostaining in the CA-treated mice brains does not provide the direct demonstration of CA on inhibiting CEBPβ by CA–CEBPβ interactions. Methods such as surface plasmon resonance (SPR) will allow for investigating the functional nature and kinetic information of CA–CEBPβ interactions.

We packaged CA in NPs to overcome the limitations induced by the poor solubility of CA and enhance the brain bioavailability of CA. The negatively charged nanoparticles showed good blood stability, but their cellular uptake was relatively low. The positively charged nanoparticles easily interact with negatively charged blood components (plasma proteins, etc.) [67], whereas, which leads to the decrease in its stability and be eliminated from the body. It is a hot issue to prepare a delivery system with the advantages of circulation stability and higher cellular uptake. Maximising drug exposures are crucial for scientific research of diseases. Nanoparticles may enter the cells through nonspecific internalisation such as phagocytosis, pinocytosis and endocytosis [68]. SBEβCD attracts more attention as a solubilizing excipient, especially for drugs with poor aqueous solubility. The negative charge located at a flexible butyl chain on the SBEβCD [69] may facilitate the interactions between SBEβCD and drug [70]; meanwhile, the flexible and anamorphous butyl chain endows higher molecular mobility, which might be beneficial to the drug permeation [71]. It has been reported that the SBEβCD-based delivery system could enhance the cellular uptake of inclusion complexed drugs in vitro and ex vivo [72]. The hydrophilic surface of SBEβCD forms a nanometre IC with the poorly water-soluble CA, improving the solubility of CA and preventing its uptake by the mononuclear phagocytic system [73], and improving drugs bioavailability [74, 75]. The hydrogen bonds between SBEβCD and CA make the IC more energetically stable [76]. In our study, the sizes of the NPs were evenly distributed, and the particle size was less than 100 nm; this facilitated the delivery of the NPs through the BBB [77] and prevented them from being filtered by the spleen [78]. The zeta potential (absolute value) was increased in the CA-*SBEβCD NPs* relative to those of the vehicle particles without CA. The results are probably due to the CA being encapsulated within the SBEβCD cavity, forming inclusion complexes through non-covalent interactions [79]. The interactions increase the density of nanostructures and enhance the stability of the nanosystem. CA-*SBEβCD NPs* exhibited sustained release behaviours without initial release burst at physiological conditions (pH 7.4). Meanwhile, the release behaviours were relatively stable in the acidic environment (pH 6.0), suggesting that the delivery system could overcome the barrier of the acid endosome, and enter the cells [80]. We verified the BBB permeability of the NPs in vitro and in vivo. The detailed transmembrane pattern and mechanism of SBEβCD delivery deserve further investigation.

## Conclusions

Our findings show that the NPs can be used as a promising brain delivery nanosystem. Preliminary studies suggested that *SBEβCD NPs* are a safe and viable option for delivering CA across the BBB. A pharmacological study of the impact of CA *SBEβCD NPs* in an AD model demonstrated that CA exerted neuroprotective effects. Oral administration of CA *SBEβCD NPs* improved the cognitive function of AD mice, inhibited proinflammatory cytokine production, and alleviated neurodegenerative processes. We observed that the increase in CEBPβ expression was related to the upregulation of the expression of NFκB target genes, contributing to the impairment of the glia-associated inflammatory response. CA treatment ameliorated glia-mediated neuronal damage under chronic inflammatory conditions and reduced Aβ deposition *via* suppressing CEBP/NFκB signalling pathway.

## Supplementary information

Supplementary Table S1

Supplementary Table S2

Supplementary Method

Supplementary Figure S1

Supplementary Figure S1. Molecular docking of CA with CEBPβ

Supplementary Figure S2

Supplementary Figure S3

Supplementary Figure S4

Supplementary Figure S5

Academic Journals Reporting Checklist

Supplementary Table S3

## Data Availability

Original western blots information is shown in Supplementary Figs. S2–S5. The datasets used and/or analysed in the present study are available from the corresponding authors.
