# Supplementary material for: Inhibition of the CEBPβ-NFκB interaction by nanocarrier-packaged Carnosic acid ameliorates glia-mediated neuroinflammation and improves cognitive function in an Alzheimer’s disease model
Source: Cell Death Dis. 2022 Apr 7;13(4):318. doi: 10.1038/s41419-022-04765-1 (PMC8989877; doi:10.1038/s41419-022-04765-1)
Supplement: Supplementary file 1 — Supplementary Table S1 [file 41419_2022_4765_MOESM1_ESM.docx]

**Supplementary Table S1 KEY RESOURCES**

| **REAGENT or RESOURCE** | **SOURCE** | **IDENTIFIER** |  | |
| --- | --- | --- | --- | --- |
| Antibodies | Supplier | Catalogue number | | Molecular Weight (kDa) |
| Rabbit anti-Aβ | Cell Signaling Technology | Cat# 8243S | | 5 |
| Mouse anti-Aβ | Sigma-Aldrich | Cat# A5213 | | 5 |
| Rabbit anti-BACE1 | Cell Signaling Technology | Cat# 5606 | | 70 |
| Rabbit anti-CD11b | Abcam | Cat# ab133357 | | 170 |
| Rabbit anti-CEBPβ | Cell Signaling Technology | Cat# 3087S | | Mouse, 36; Human, 45 |
| Mouse anti-CEBPβ | Santa Cruz Biotechnology | Cat# sc-7962 | | 45 |
| Rabbit anti-COX2 | Cell Signaling Technology | Cat# 12282S | | 74 |
| Rabbit anti-GAPDH | Kang Chen | Cat# KC-5G5 | | 35 |
| Mouse anti-GFAP | Cell Signaling Technology | Cat# 3670 | | 50 |
| Rabbit anti-Iba1 | Wako | Cat. No.016-20001 | | 17 |
| Mouse anti-Iba1 | Wako | Cat. No.016-26721 | | 17 |
| Rabbit anti-IL-1β | Cell Signaling Technology | Cat# 12426S | | 17, 31 |
| Rabbit anti-IL6 | Cell Signaling Technology | Cat# 12912S | | 24 |
| Rabbit anti-Lamin B1 | Abcam | Cat# ab16048 | | 66 |
| Rabbit anti-NFκB p65 | Cell Signaling Technology | Cat# 8242S | | 65 |
| Rabbit anti-NLRP3 | Cell Signaling Technology | Cat# 15101S | | 110 |
| Rabbit anti-PSD95 | Cell Signaling Technology | Cat# 3450S | | 95 |
| Rabbit anti-Synapsin 1 | Cell Signaling Technology | Cat # 5297 | | 77 |
| Rabbit anti-Synaptophysin | Abcam | Cat# ab32127 | | 34 |
| Rabbit anti-TNFα | Santa Cruz Biotechnology | Cat# sc-52746 | | 26 |
| Critical Commercial Assays | Supplier | Catalogue number | |  |
| Aβ1-40 Human ELISA Kit | Invitrogen, Thermo Scientific | Cat# KHB3482 | |  |
| Aβ1-42 Human ELISA Kit | Invitrogen, Thermo Scientific | Cat# KHB3442 | |  |
| Nuclear Extract Kit | Active Motif | Cat# 40010 | |  |
| Annexin V-FITC Apoptosis Detection Kit | Cell Signaling Technology | Cat# 6592 | |  |
| V-PLEX Human IL-1β Kit | Meso Scale Discovery | Cat# K151QPD | |  |
| V-PLEX Human IL-6 Kit | Meso Scale Discovery | Cat# K151QXD | |  |
| MitoProbe™ JC-1 Assay Kit | Thermo Scientific | Cat# M34152 | |  |
| NFκB Activation Assay Colorimetric Kits | Active Motif | Cat# 40096 | |  |
| V-PLEX Human TNF-α Kit | Meso Scale Discovery | Cat# K151QWD | |  |
| In Situ Cell Detection Kit | Roche Diagnostics GmbH | Cat# 11684817910 | |  |
