# Supplementary material for: Inhibition of the CEBPβ-NFκB interaction by nanocarrier-packaged Carnosic acid ameliorates glia-mediated neuroinflammation and improves cognitive function in an Alzheimer’s disease model
Source: Cell Death Dis. 2022 Apr 7;13(4):318. doi: 10.1038/s41419-022-04765-1 (PMC8989877; doi:10.1038/s41419-022-04765-1)
Supplement: Supplementary file 2 — Supplementary Table S2 [file 41419_2022_4765_MOESM2_ESM.docx]

| **Gene** | **Sequences (5’-3’)** | **Gene Full Name** |
| --- | --- | --- |
| NFκB | Forward: CAGTGACGGGGGATGTGAAGA | nuclear factor-κB |
|  | Reverse: CAGTGACGGGGGATGTGAAGA |  |
| CEBPβ | Forward: CGCCGCCTTTAGACCCA | CCAAT-enhancer binding protein-β |
|  | Reverse: CGCTCGTGCTCGCCAAT |  |
| IL-6 | Forward: AAGCCAGAGTCCTTCAGAGAG | Interleukin-6 |
|  | Reverse: TTGGATGGTCTTGGTCCTTAG |  |
| TNFɑ | Forward: TGAACTTCGGGGTGATCGGT | tumor necrosis factor-α |
|  | Reverse: TGTGAGTGTGAGGGTCTGGG |  |
| β-actin | Forward: TTCAACACCCCAGCCATGT | beta-actin |
|  | Reverse: TGTGGTACGACCAGCGGCATAC |  |

**Supplementary Table S2 qRT-PCR primer list**
