# Supplementary material for: Inhibition of the CEBPβ-NFκB interaction by nanocarrier-packaged Carnosic acid ameliorates glia-mediated neuroinflammation and improves cognitive function in an Alzheimer’s disease model
Source: Cell Death Dis. 2022 Apr 7;13(4):318. doi: 10.1038/s41419-022-04765-1 (PMC8989877; doi:10.1038/s41419-022-04765-1)
Supplement: Supplementary file 3 — Supplementary Method [file 41419_2022_4765_MOESM3_ESM.docx]

**Molecular docking**

Two-dimensional structure of carnosic acid (CA) and Sulfobutyl ether-beta-cyclodextrin (SBEβCD) was downloaded from PubChem database (https://pubchem.ncbi.nlm.nih.gov), and were converted from SDF format into three-dimensional (3D) structures using ChemDraw 20.0 and Chem3D 20.0 software. After minimized the energy, the 3D structures of CA and SBEβCD were uploaded to YINFO Technology workstation (http://www.yinfotek.com/) to pretreat for molecular docking (Code availability). The interaction of CA and SBEβCD was simulated using molecule-molecule docking module and calculations were performed with Autodock Vina. The entire binding site was noted in a grid of points spaced at 16 × 16 × 16 using a grid space of 1 Å (centers of grid box: x = -1.813; y = 0.276; z = 0.84). After docking simulation, the conformations of CA-SBEβCD interaction at the status of the highest negative binding free energy (-kiloJoules/moL) were selected as the best pose of CA-SBEβCD binding. The best conformation was visualized and analyzed. The hydrogen bonds interactions between CA and SBEβCD were indicated by blue dotted line.
