# Supplementary material for: Inhibition of the CEBPβ-NFκB interaction by nanocarrier-packaged Carnosic acid ameliorates glia-mediated neuroinflammation and improves cognitive function in an Alzheimer’s disease model
Source: Cell Death Dis. 2022 Apr 7;13(4):318. doi: 10.1038/s41419-022-04765-1 (PMC8989877; doi:10.1038/s41419-022-04765-1)
Supplement: Supplementary file 4 — Supplementary Figure S1 [file 41419_2022_4765_MOESM4_ESM.docx]

**Supplementary Figure S1. Molecular docking assays suggest the interactions of CA with CEBPβ.** The 3D structure of CA was downloaded from Zinc database (https://zinc.docking.org). The crystal structure of CEBPβ was retrieved from the Protein Data Bank (http://www.rcsb. org). Docking simulations were conducted using Autodock Tool ver1.5.6. The water molecules were removed and polar hydrogens were added. The typical binding region was selected using Autodock Vina ver1.1.2, Autodock tools were applied to conduct affinity grid maps. The grid conditions were 40 × 100 × 50 points with a grid spacing of 1.0 Å (centers of grid box: x = 75.031; y = 53.455; z = 78.023). Docked sites were analyzed with rankings according to the binding energies. The best poses were determined for hydrogen bonding, hydrophobic and van derWaals interactons interactions using pyMOL Program ver 1.6 (Code availability). CA is connected to CEBPβ through hydrogen bonding between the O atom with OH of Alanine (Ala) 292 (3.0 Å), and the O atom with the H-N of Asparagine (Asn) 296 (3.4 Å). The binding affinity was -25.12 KJoule/mol. Yellow dotted line indicates the residues interacting with CA via hydrogen bonds.
