# Supplementary figures and images for: Inhibition of the CEBPβ-NFκB interaction by nanocarrier-packaged Carnosic acid ameliorates glia-mediated neuroinflammation and improves cognitive function in an Alzheimer’s disease model
Source: Cell Death Dis. 2022 Apr 7;13(4):318. doi: 10.1038/s41419-022-04765-1 (PMC8989877; doi:10.1038/s41419-022-04765-1)

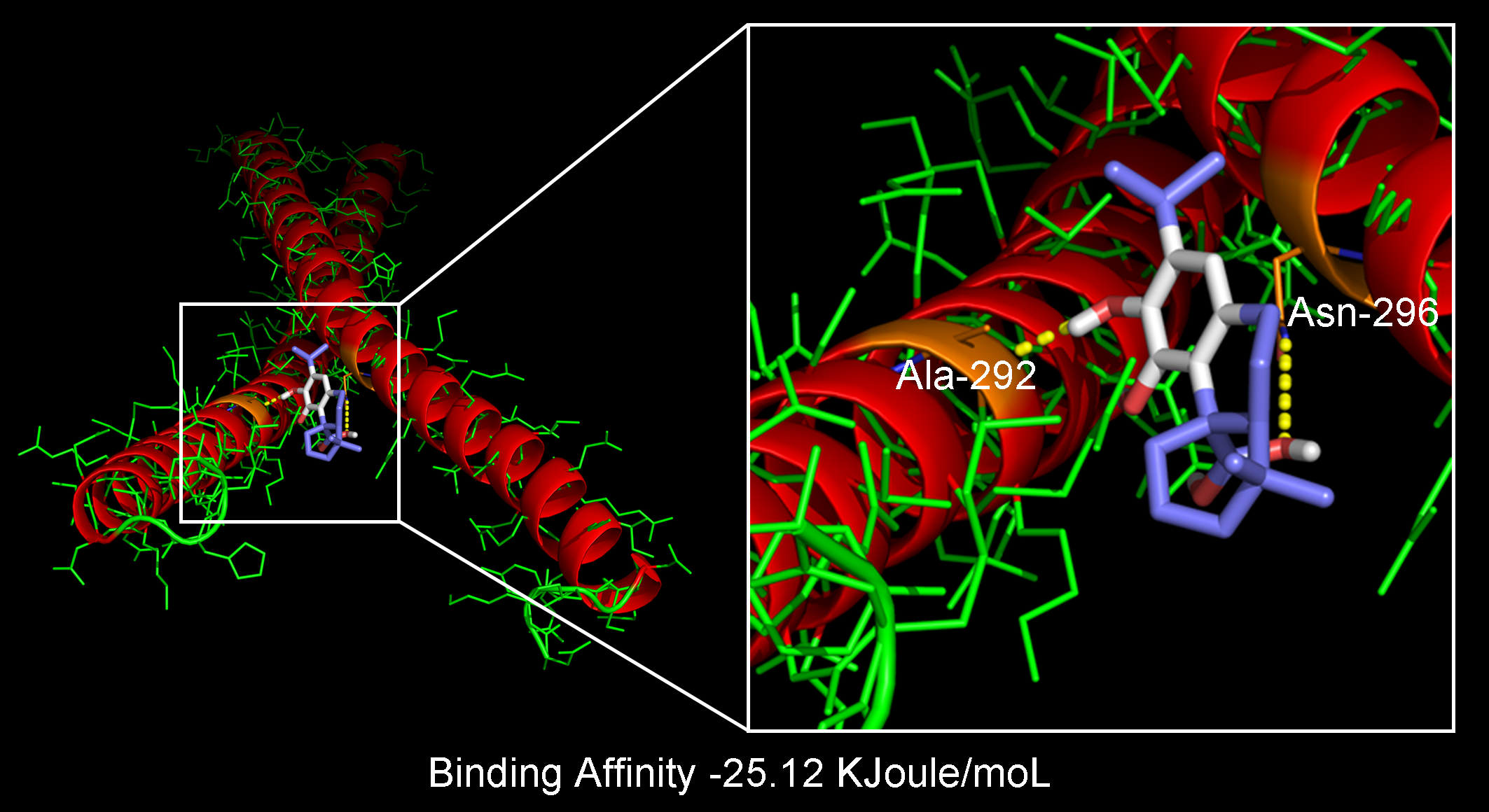

Supplement: Supplementary file 5 — Supplementary Figure S1. Molecular docking of CA with CEBPβ [file 41419_2022_4765_MOESM5_ESM.tif]

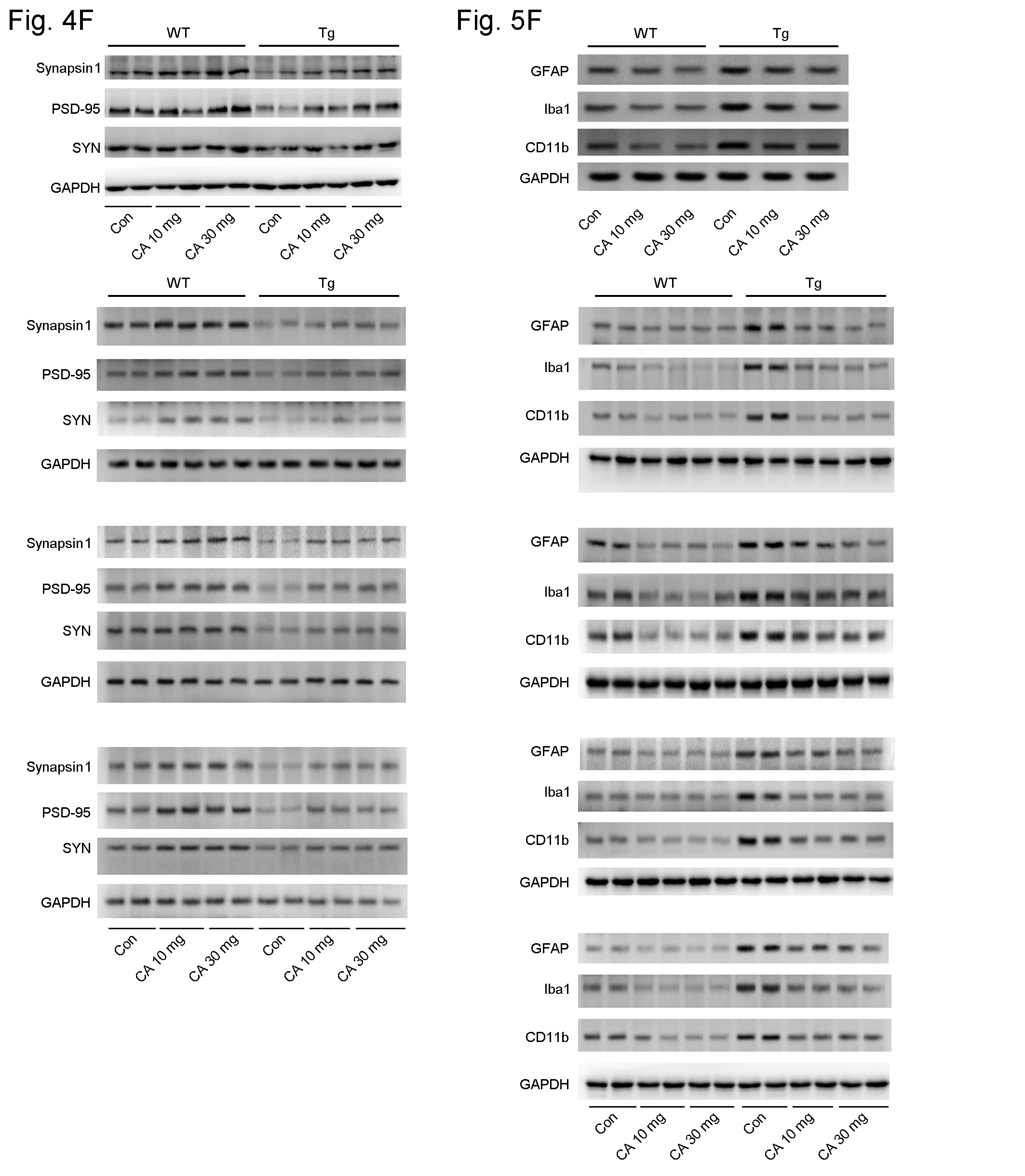

Supplement: Supplementary file 6 — Supplementary Figure S2 [file 41419_2022_4765_MOESM6_ESM.tif]

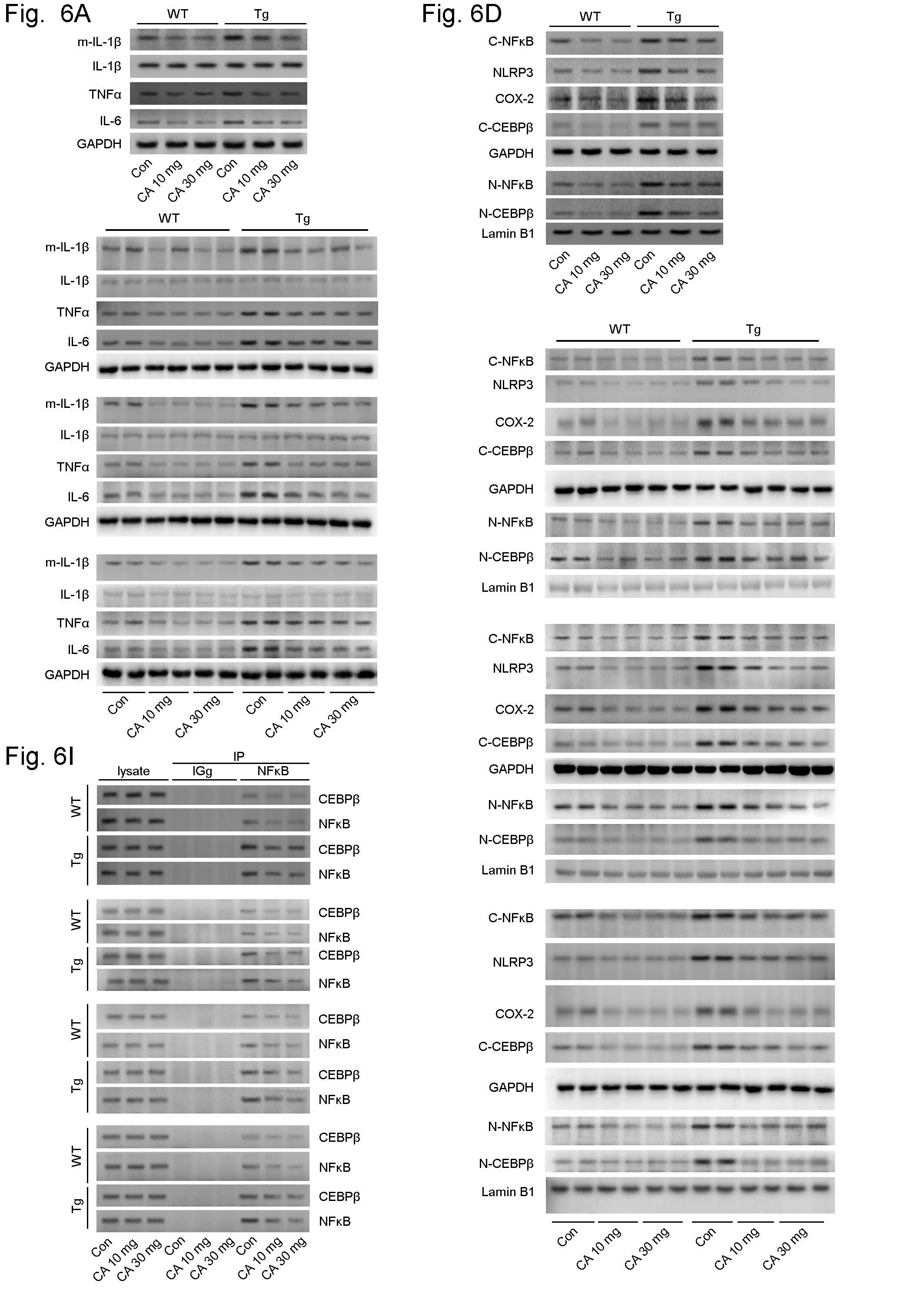

Supplement: Supplementary file 7 — Supplementary Figure S3 [file 41419_2022_4765_MOESM7_ESM.tif]

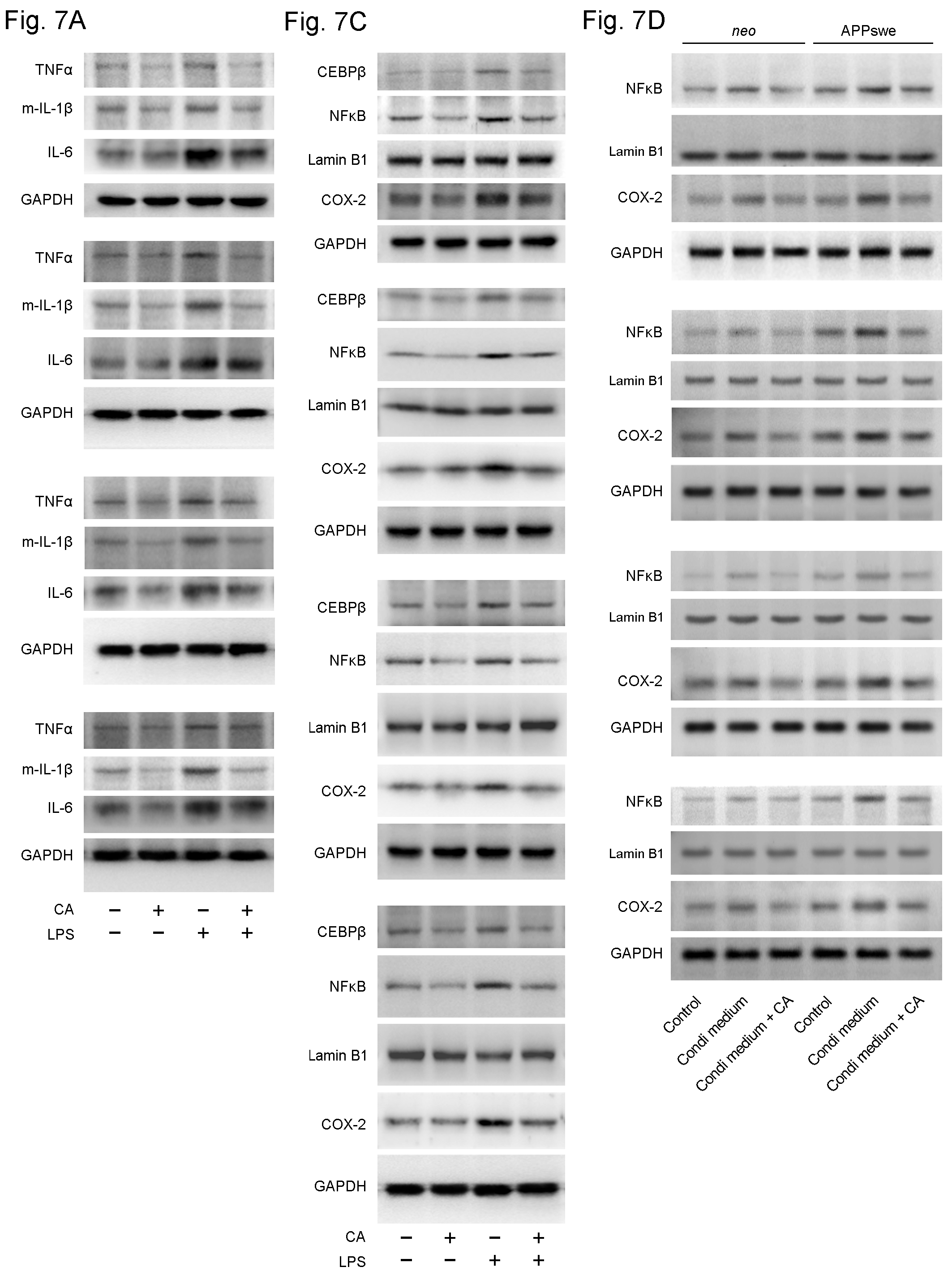

Supplement: Supplementary file 8 — Supplementary Figure S4 [file 41419_2022_4765_MOESM8_ESM.tif]

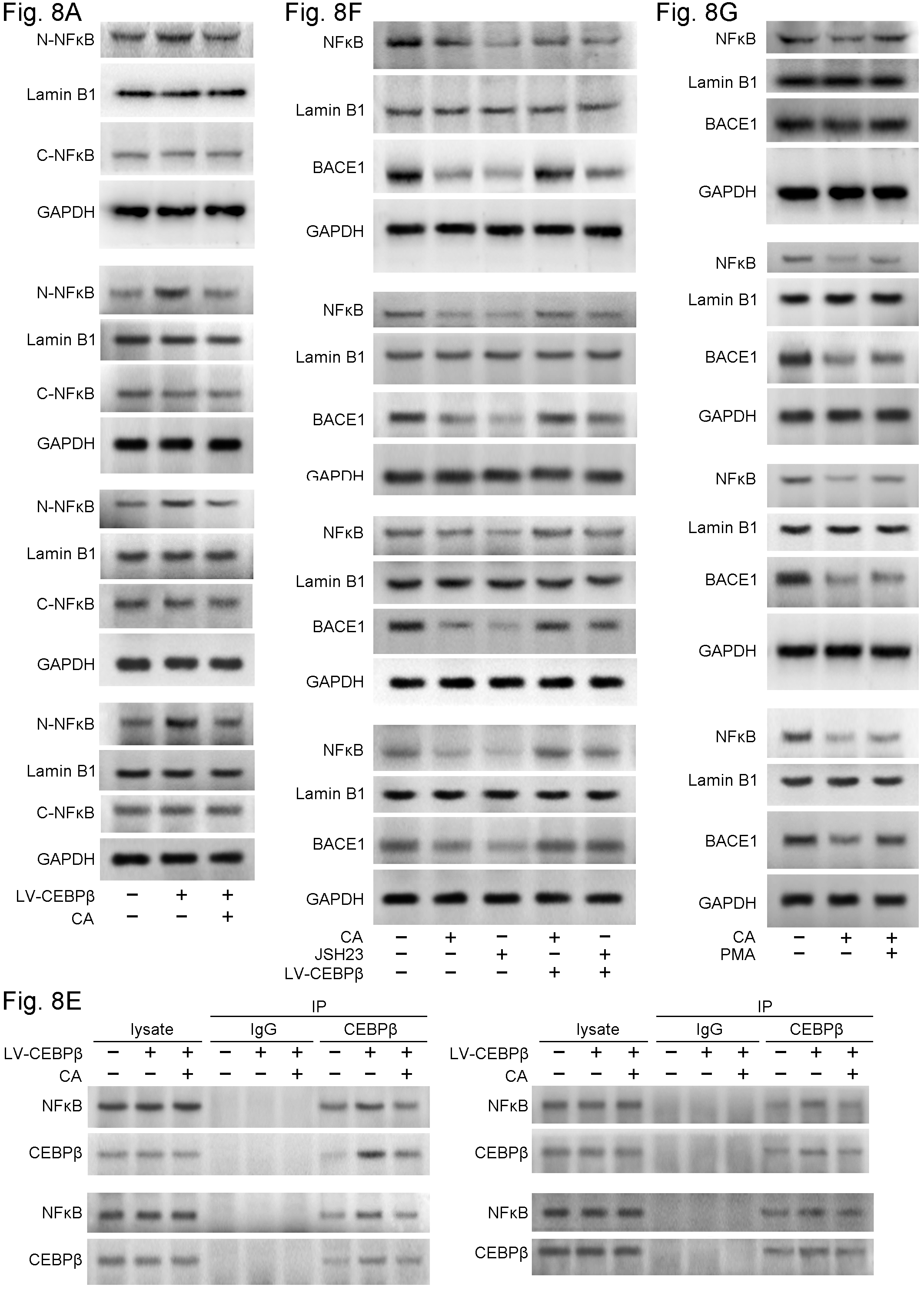

Supplement: Supplementary file 9 — Supplementary Figure S5 [file 41419_2022_4765_MOESM9_ESM.tif]
