# Supplementary material for: Inhibition of the CEBPβ-NFκB interaction by nanocarrier-packaged Carnosic acid ameliorates glia-mediated neuroinflammation and improves cognitive function in an Alzheimer’s disease model
Source: Cell Death Dis. 2022 Apr 7;13(4):318. doi: 10.1038/s41419-022-04765-1 (PMC8989877; doi:10.1038/s41419-022-04765-1)
Supplement: Supplementary file 11 — Supplementary Table S3 [file 41419_2022_4765_MOESM11_ESM.docx]

**Table S3 The optimization design and characteristics of CA-*SBEβCD NPs***

| **The orthogonal design of CA-*SBEβCD NPs*** | | | | | | | |
| --- | --- | --- | --- | --- | --- | --- | --- |
| **CA/SBEβCD**  **(molar ratio)** | **PEG400**  **(v/v, %)** | | **Tween80**  **(v/v, %)** | **Poloxamer407**  **(m/v, %)** | | **EE**  **(%)** | **LE**  **(%)** |
| 1:0.5 | 0.5 | | 0.25 | 2.5 | | 81.73±1.65 | 7.42±1.14 |
| 1:1 | 0.5 | | 0.5 | 5 | | 93.66±0.73 | 6.64±0.45 |
| 1:2 | 0.5 | | 1.25 | 12.5 | | 93.51±1.34 | 4.07±0.16 |
| 1:1 | 1.25 | | 0.25 | 12.5 | | 89.97±1.03 | 4.68±0.20 |
| 1:2 | 1.25 | | 0.5 | 2.5 | | 93.29±3.31 | 4.39±0.32 |
| 1:0.5 | 1.25 | | 1.25 | 5 | | 91.90±1.97 | 4.64±0.54 |
| 1:2 | 2.5 | | 0.25 | 5 | | 81.22±1.26 | 3.13±0.26 |
| 1:0.5 | 2.5 | | 0.5 | 12.5 | | 81.07±2.63 | 3.34±0.23 |
| 1:1 | 2.5 | | 1.25 | 2.5 | | 74.42±4.17 | 3.45±0.44 |
| **The characteristics of CA-*SBEβCD NPs*** | | | | | | | |
| **Sample** | **Intensity size (nm)** | **Zeta potential (mV)** | | | **PDI** | **EE**  **(%)** | **LE**  **(%)** |
| **Vehicle *NPs*** | 24.53±5.14 | -5.73±2.12 | | | 0.243±0.093 | — | — |
| **CA-loaded *NPs*** | 36.42±083 | -11.54±1.45 | | | 0.264±0.004 | 93.66±0.73 | 6.64±0.45 |
